# Multidimensional dynamic characterization and decoding of finger movements using magnetoencephalography

**DOI:** 10.1162/IMAG.a.1182

**Published:** 2026-03-30

**Authors:** Yu Zheng, Xu Wang, Li Zheng, Hongtao Zhang, Fan Wang, Yan Zhuo

**Affiliations:** State Key Laboratory of Cognitive Science and Mental Health, Institute of Biophysics, Chinese Academy of Sciences, Beijing, China; University of Chinese Academy of Sciences, Beijing, China; State Key Laboratory of Mechanical System and Vibration, School of Mechanical Engineering, Shanghai Jiao Tong University, Shanghai, China

**Keywords:** magnetoencephalography, finger movement, source level, motor execution

## Abstract

The similarity of neural activity in finger movements poses challenges for accurate decoding using many non-invasive imaging techniques. Magnetoencephalography (MEG), with its relatively high spatial resolution, offers the potential to capture the underlying dynamic neural differences. In this study, we recorded MEG signals during single extension movements of the right-hand fingers, examining the time-varying cortical activation patterns across different frequency bands and their contribution to decoding finger movements. Our results demonstrate that signals below 8 Hz not only enable effective movement classification but also reveal millisecond-scale neural activation patterns in the sensorimotor cortex. Furthermore, incorporating the spatiotemporal dynamics of neural activity may enhance decoding performance for fine motor control. These findings highlight the value of integrating temporal, frequency, and spatial dimensions in studying motor neural activity and underscore MEG’s potential for broader applications in movement-related neurophysiology and brain–computer interface research.

## 1 Introduction

Movement execution involves the integration of sensory and motor information processing across widespread brain regions. The primary sensorimotor cortex plays a central role in movement planning (Li et al., 2015), control, inhibition (Ebbesen & Brecht, 2017; Shenoy et al., 2013), and the regulation of movement states (Gale et al., 2021; Schneider, 2020). Traditionally, its functional organization has been considered contralateral and somatotopically structured, as depicted in the Homunculus model (Penfield & Rasmussen, 1950; Roux et al., 2020). However, recent studies indicate that movement control may involve broader and more complex cortical circuits beyond this classical representation (Gordon et al., 2023; Graziano, 2016).

In human motor research, decoding neural activity in the sensorimotor cortex using neuroimaging techniques is essential for elucidating the neural mechanisms underlying movement. Advances in motor functions rehabilitation and brain–computer interface (BCI) systems have further driven interest in decoding limb movement-related neural activity through invasive or non-invasive imaging modalities (Lorach et al., 2023; Tam et al., 2019). Among these, decoding finger movements has gained significant attention due to the critical role of fingers in daily activities and their exceptional functional dexterity. Researchers have successfully decoded different finger movements and hand gestures using techniques such as electrocorticogram (ECoG) (Branco et al., 2017; Merino et al., 2023), electroencephalogram (EEG) (Alazrai et al., 2019; Xu et al., 2021; Yoshimura et al., 2017), and functional near-infrared spectroscopy (fNIRS) (Khan et al., 2021), achieving promising results. Recently, EEG-based BCI systems capable of controlling robotic arms through decoded finger movements have also been successfully demonstrated (Ding et al., 2025), further underscoring the significance of research on finger movement decoding.

Movement-related neural activity in the sensorimotor cortex evolves across multiple frequency bands over time. δ- and θ-band activities are consistently observed during movement execution (Körmendi et al., 2021; Wang et al., 2024) and may exhibit movement-related phase-locking (Popovych et al., 2016). Recent studies have demonstrated strong correlations between these low-frequency oscillations and muscle activity during finger movements, with both bands accurately localized to the primary motor cortex (Huang et al., 2024). Moreover, delta-band power modulation often emerges at the stage of movement intention, preceding actual motor execution (Noel et al., 2025). In the α and β bands, event-related desynchronization (ERD) occurs during movement preparation and execution, followed by event-related synchronization (ERS) after movement termination. This modulation, known as the sensorimotor rhythm, is thought to reflect transient disruption and subsequent reactivation of the resting state in the sensorimotor cortex (Cheyne, 2013; Pfurtscheller & Lopes da Silva, 1999). Additionally, an increase in power within the high-γ-band (60–90 Hz), termed movement-related gamma synchronization (MRGS), has been observed at movement onset (Heinrichs-Graham et al., 2018; Pfurtscheller et al., 2003). These features have been widely used features in finger movement decoding studies (Ding et al., 2025; Iturrate et al., 2018; Jerbi et al., 2011; Xu et al., 2021).

Beyond temporal features, the spatial localization of movement-related neural activity on the cortex is another critical aspect of decoding. Multiple studies have demonstrated that incorporating spatial information extracted via source imaging techniques can significantly enhance classification performance (Edelman et al., 2016; Handiru et al., 2017). However, accurately localizing neural activity for finger movements remains challenging for non-invasive imaging techniques, as the somatotopic representation of individual fingers on the sensorimotor cortex is separated by only a few millimeters (Ejaz et al., 2015; Huber et al., 2020).

Currently, magnetoencephalography (MEG), a non-invasive neuroimaging technique, has demonstrated significant potential in hand movement decoding studies. Like EEG, MEG provides millisecond-level temporal resolution, enabling the precise tracking of whole-brain neural dynamics. However, MEG outperforms EEG in spatial localization due to its immunity to volume conduction effects, which often blur signals in EEG recordings (Baillet, 2017). Studies have been reported that MEG can achieve a spatial resolution of 3–5 mm (Barratt et al., 2018), allowing it to capture neural activity from highly localized cortical regions with greater precision. Moreover, the absence of signal smearing by brain tissues facilitates more accurate source imaging, making MEG particularly advantageous for reconstructing neural activity at the cortical level. Additionally, research suggests that MEG is less susceptible to electromyographic contamination compared with EEG (Siems et al., 2016), allowing for more reliable recording of neural oscillations in higher frequency bands. These advantages enable MEG to capture the dynamic neural processes underlying movement, making it especially well suited for decoding fine and rapid motor actions.

MEG has shown strong potential for decoding finger and hand movements, outperforming EEG in similar tasks (Quandt et al., 2012). Previous studies have successfully classified finger presses, hand gestures, and both real and imagined movements using MEG (Bu et al., 2023; Quandt et al., 2012; Zubarev et al., 2024). Additionally, MEG has been effective in decoding continuous movement parameters, such as hand trajectory and movement speed (Jerbi et al., 2011; Kim et al., 2023; Waldert et al., 2008). Based on its unique advantages and previous research, MEG may have the potential to simultaneously characterize the cortical neural dynamics of finger movements across both temporal and spatial dimensions, which is a key focus of our work.

In this study, we aim to characterize cortical representations of multi-band neural activity in the sensorimotor cortex during individual finger movements with high temporal and spatial resolution, and to evaluate their contributions to decoding performance. MEG was employed to measure neural signals during single extension movements of the right thumb, index, middle, and little fingers. Time-resolved and spatially distributed features of δ, θ, α, β, γ and high-γ bands within the contralateral sensorimotor cortex were extracted and applied to four-class classification. We systematically examined how temporal, spatial, and frequency-domain information captured by MEG contributes to the decoding of motor representations and reflects the dynamic neural processes underlying voluntary movement. Finally, we mapped the temporal evolution of these cortical patterns onto the contralateral sensorimotor cortex to visualize the dynamic separation of neural representations associated with different fingers.

## 2 Methods

### 2.1 Participants

Eighteen healthy right-handed participants (10 females; age 24.78 ± 3.56 years) participated in this study. None of them had known neurological or muscular disorders. The study was approved by the ethics committee of the Institute of Biophysics, Chinese Academy of Sciences (approval number 2023-IRBH-001). All participants were informed of the experimental procedures prior to data collection and provided written informed consent.

### 2.2 Experimental paradigm

**Fig. 1.**
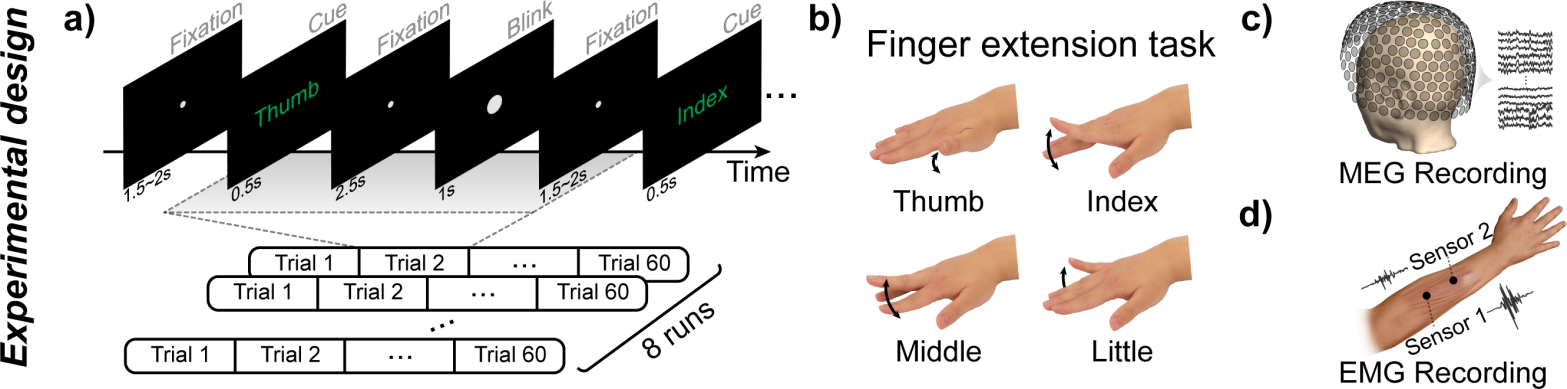
Experimental design. (a) Cue-based experimental paradigm. In each trial, participants executed a single, brisk extension movement of the target finger in response to a visual cue. During the period of enlarged size gaze point, participants were allowed to perform one to two natural blinks, ensuring that blinking was restricted to this phase while helping maintain focus and attentional engagement. (b) Four finger extension tasks. (c) Magnetoencephalography (MEG) recording by a full-head superconducting quantum interference device (SQUID) system. (d) The location of electromyogram (EMG) electrodes.

During the experiment, participants were seated comfortably with their arms resting on armrests, palms down, while viewing a semi-transparent screen with a resolution of 1024 × 768 pixels positioned 751 mm from their eyes. Participants performed a series of right-hand finger movements and blinking tasks in response to on-screen visual cues. The experimental paradigm is illustrated in Figure 1a and b. The finger movement tasks involved the thumb, index, middle, and little fingers, while the ring finger was excluded because most participants found it difficult to perform isolated extensions with this finger. Prior to the main experiment, participants practiced isolated finger extensions while monitoring their electromyogram (EMG) signals to ensure comparable movement amplitudes and to minimize visible co-movements of non-target fingers. During the main experiment, they were instructed to replicate the same movement pattern established during practice.

Each trial began with a white gaze point displayed at the center of the screen for 1 s, during which participants were instructed to maintain attention. This was followed by a green visual cue displaying the name of one of four target fingers. The cue was presented for 500 ms, and participants were required to perform a single brisk extension movement of the corresponding finger. During the task, all body parts except the target finger remained relaxed, and the finger was required to return to a relaxed state immediately after the movement.

After the cue disappeared, the gaze point remained on the screen for 2.5 s. Participants were asked to maintain their gaze throughout the presentation of the above picture. The gaze point then briefly increased in size for 1 s, during which participants were allowed to perform 1–2 times natural blinks. This step was designed to minimize blink-related artifacts and help maintain participants’ attention. Following this, the gaze point returned to its original size, and participants resumed a relaxed whole-body state. Each trial was followed by an inter-trial interval randomly set between 0.5 and 1 s, resulting in a total trial duration of 5.5–6 s. Each run consisted of 60 trials (15 trials for each task), with task order randomized. Each participant performed 8 runs, with a 2 min rest period between runs to prevent fatigue.

Visual stimuli were generated and presented using MATLAB, Psychtoolbox version 3 (Kleiner et al., 2007) (http://psychtoolbox.org). A 60 Hz projector system (NEC, NP63+) located outside the shielded room was used to present all visual stimuli.

### 2.3 Data acquisition

MEG data were recorded using a full-head system (MISL-CTF DSQ-3500, Vancouver, BC, Canada) comprising 275 first-order radial gradiometers (excluding 5 defective channels) with a sampling rate of 1200 Hz (Fig. 1c). The MEG system was housed in a magnetically shielded room (Vacuumschmelze GmbH & Co. KG, Hanau, Germany) to reduce ambient magnetic interference. Participants’ head positions within the helmet were continuously tracked using three head position indicator (HPI) coils placed at the nasion and the right and left preauricular points. These head position data were used to realign the MEG sensor array with individual MR images. Participants were instructed to keep head movements below 5 mm with the assistance of an additional holder.

To detect the participant’s finger movements, two Ag/AgCl electrodes were placed on the extensor digitorum muscle to record EMG signals, as shown in Figure 1d. The passive electrodes were connected to an BrainAmp DC amplifier (Brain Products GmbH, Gilching, Germany) for data acquisition at a sampling rate of 1000 Hz.

At the end of simultaneous MEG and EMG acquisition, T1-weighted brain magnetic resonance images of the participants were acquired using a 3T MRI system (MAGNETOM Prismafit, Siemens, Erlangen, Germany) with a resolution of 1 × 1 × 1 mm.

### 2.4 Data processing

The analysis comprised four main stages. First, EMG signals were preprocessed and subjected to four-class classification to evaluate the consistency within trials and the discriminability across tasks from a behavioral perspective. Second, MEG signals were projected onto the source space constrained to the left precentral and postcentral gyri. Both temporal and spatial analyses were performed to characterize task-related dynamics across six distinct frequency bands. Third, the spatiotemporal representations obtained from each frequency band were used as features for four-class classification with a support vector machine (SVM), aiming to assess their ability to discriminate between finger movements. Finally, the analysis was extended to cover a broader region of the contralateral sensorimotor cortex and a longer time span using classification method, to characterize the differences between four tasks.

#### 2.4.1 EMG data processing

The EMG data were resampled to 1200 Hz to match MEG data. The raw signals were band-pass filtered between 20 and 450 Hz, full-wave rectified, and then smoothed using a 50 Hz low-pass filter. The movement initiation threshold was derived for each trial using the following equation (Hodges & Bui, 1996; Solnik et al., 2010):

$$\begin{array}{c} T=\mu +k\sigma ,\; \end{array}$$ (1)

where μ and σ depicted the mean and standard deviation of the epoch, k took the value of 2. The first moment of exceeding the threshold was marked as the movement onset. The interval between this time point and the onset of the visual cue was defined as the reaction time and used to align the 0 ms in subsequent MEG analyses.

Using the same threshold, the last time point at which the EMG signal dropped below the threshold within each trial was identified as the movement offset. The interval between the onset and offset represented the movement time. Together, reaction time and movement time reflected both the consistency of task execution within trials and performance differences across tasks.

Finally, the preprocessed EMG signals were segmented from 1.0 s before to 2.0 s after the movement onset and used as features for four-class classification with a one-versus-rest (OVR) SVM. This analysis aimed to assess task discriminability based on muscle activity, reflecting differences in motor execution across tasks.

#### 2.4.2 MEG and MRI data pre-processing

The MEG data were band-pass filtered from 0.5 to 90 Hz and segmented from -2.0 s to 2.0 s around movement onset for each trial. Epochs containing MEG or EMG artifacts, or those in which participants failed to perform the task correctly (e.g., excessively long reaction times or incorrect finger movements), were excluded from further analysis. For each participant, at least 100 valid trials per task were required to be retained. Notably, this procedure was performed prior to EMG analysis. Two participants were excluded from the study because excessive trial rejection resulted in insufficient remaining data. Artifacts arising from eye movements and cardiac activity were mitigated using independent component analysis (ICA). The preprocessed MEG data were then downsampled to 300 Hz and divided into six frequency bands following previous movement-related studies (Ding et al., 2025; Heinrichs-Graham et al., 2018; Noel et al., 2025): δ (0.5–4 Hz), θ (4–8 Hz), α (8–13 Hz), β (13–30 Hz), γ (30–60 Hz), and high-γ (60–90 Hz), which were used for subsequent analyses. All this processing was implemented with custom programs developed with the Fieldtrip toolbox (Donders Institute for Brain, Cognition and Behaviour, https://www.fieldtriptoolbox.org) (Oostenveld et al., 2011) on MATLAB (MathWorks Inc.).

Head position data recorded by the HPI coils were segmented into epochs aligned with movement initiation times. The head positions from all trials were averaged to determine a mean head position. This mean position was then used to adjust the co-registration of the MEG sensor array with the participant’s head, ensuring accurate alignment for subsequent analyses.

Individual T1-weighted structure MR images were acquired and aligned to the MEG sensor array. Cortical surface was reconstructed using FreeSurfer (http://surfer.nmr.mgh.harvard.edu) (Fischl, 2012), and the gray–white matter boundary surface was subsequently downsampled to 15684 vertices (7842 per hemisphere) with Connectome Workbench (https://www.humanconnectome.org/software/connectome-workbench) (Glasser et al., 2016), serving as locations for source reconstruction. Each participant’s surface was aligned with MNI152 template and parcellated according to the “Desikan–Killiany” cortical atlas (Desikan et al., 2006).

#### 2.4.3 Source level analysis

To extract task-related neural activity with higher spatial resolution and reduced noise, preprocessed MEG signals were projected onto vertices within the contralateral primary sensorimotor cortex using linearly constrained minimum variance (LCMV) beamforming (Van Veen et al., 1997). The procedure followed the implementation described by O’Neill et al. (2019). The data covariance matrix was computed across the entire experimental period. For each vertex, the reconstructed signal was projected along the direction of maximal variance, as determined by eigenvalue decomposition.

To characterize the temporal dynamics of the source-reconstructed signals, time–frequency responses (TFRs) were computed using Morlet wavelet analysis with four cycles, yielding a temporal resolution of 100 ms.

In addition, principal component analysis (PCA) was performed on the source-reconstructed signals within the region-of-interest (ROI, contralateral primary sensorimotor cortex), and components explaining over 70% of the total variance were retained to derive task-related temporal signals that were independent of precise spatial information.

Conversely, to obtain the spatial activation patterns of task-related neural activity, the activation index (*A*) was computed at each vertex according to Equation (2) following the method described by O’Neill et al. (2019):

$$\begin{array}{c} A\left(i\right)=\;\frac{\text{w}{\text{C}}_{s}\left(i\right){\text{w}}^{T}-\;\text{w}{\text{C}}_{b}\left(i\right){\text{w}}^{T}}{\text{w}{\text{C}}_{s}\left(i\right){\text{w}}^{T}+\;\text{w}{\text{C}}_{b}\left(i\right){\text{w}}^{T}}, \end{array}$$ (2)

where w denotes beamformer weights for each vertex and ${\text{C}}_{s}\left(i\right)$ and ${\text{C}}_{b}\left(i\right)$ denote the covariance matrices of all channels during the task and baseline periods for the $i^{th}$ trial, respectively. The interval from –2.0 to –1.5 s was defined as the baseline period, and –1.0 to 2.0 s as the task period. The 1.0 s pre-movement window was longer than the reaction time of all participants, ensuring that task-related neural activity following the visual cue was fully captured for every participant. The covariance matrix and activation index were computed separately for each trial, and the resulting *A* was then averaged across trials.

Two task–segment lengths were used to compute the activation index in the analyses. (1) The full-epoch activation index (*A*-epoch) map was derived using the task period from −1 s to 2 s relative to movement onset. (2) The short-window activation index (*A*-window) maps were obtained by dividing the epoch into nonoverlapping 50-ms windows, within which the *A* was computed separately for each time window.

#### 2.4.4 Classification

The classification analyses examined task-related neural activity from three perspectives—frequency, space, and time—to assess its ability to differentiate the four finger movements. For each participant, classification was performed separately for each frequency band using a linear OVR SVM implemented in the LIBSVM toolbox (Chang & Lin, 2011).

First, to evaluate the temporal and spatial discriminability of task-related activation patterns across different frequency bands, two complementary time-resolved decoding analyses were performed. Specifically, we performed four-class classification using both *A*-window maps (window-wise) and the source-reconstructed signals (point-wise) within ROI from all frequency bands. In the window-wise analysis, *A*-window map from each window was treated as an independent feature sample, whereas in the point-wise analysis, each time point served as a separate input. The classification was implemented using the MVPA-Light toolbox (Treder, 2020) with an SVM classifier.

Second, to investigate how the utilization of cortical spatial information influences decoding performance, three classification analyses were conducted using spatial features extracted at different levels of spatiotemporal resolution:

1. The PCA components retained for each single trial (Section 2.4.3) were used as features by directly inputting their time series during the task period (–1 to 2 s) into the classifier. This method was referred to as the PCA-based classification, where the spatial information represented the entire ROI.
2. For each task, the vertices showing the maximum and minimum activation indices in the *A*-epoch map were identified as the most task-related activation locations. Two vertices were selected per task, resulting in a total of eight vertices across the four tasks. Since the temporal trends of neural activity varied across frequency bands, both the maximum and minimum activation indices were used to represent task-related activation patterns. For all trials, the source-reconstructed time series at these vertices were used as features for classification. This method, referred to as the *A*-epoch-based classification, aimed to capture spatial information corresponding to the most task-relevant neural activity throughout the entire task period. It refined the spatial resolution of the extracted features compared with the PCA-based method.
3. To incorporate the temporal dynamics of task-related spatial patterns, the vertices showing the maximum and minimum activation indices in each window of the *A*-window maps were identified for all four tasks, yielding eight locations per window. The 50 ms source-reconstructed time series at these vertices were then extracted for each corresponding time window. Finally, the time series from all 60 windows were concatenated in temporal order to form the feature set, matching the dimensionality of method (2). This method, referred to as the *A*-window-based classification, introduced time-resolved spatial information with a 50 ms resolution. It allowed us to evaluate how extending spatial features along the temporal dimension contributes to decoding performance.

Finally, we further extended the spatial and temporal information contained in the classification features to improve decoding performance. Based on the results of the window-wise classification (Section 2.4.4 Step 1), we first selected the time windows that showed decoding accuracies significantly higher than the 25% chance level for each frequency band. For each selected window, the vertices corresponding to the maximum and minimum activation indices were extracted following the procedure described in Step 2(3). These vertices formed a set of virtual channels in source space (window number × 4 tasks × 2), representing the task-relevant spatial activation patterns that best captured task differences. The reconstructed time series from these virtual channels were then extracted, and the segments corresponding to the decodable periods determined from the point-wise classification were used as classifier inputs. This ensured that only the signal components most likely to contain task-specific information were retained.

To evaluate the effectiveness of this multi-dimensional feature integration, two classification schemes were implemented: (1) Single-window classification, using only time series from the virtual channels corresponding to single decodable time window (eight channels in total); and (2) Window-combined classification, using time series from all virtual channels across all decodable windows as classifier inputs.

In this section, classification was performed using a 5-fold cross-validation procedure, with the model trained on 80% of the data and tested on the remaining 20% of trials. Activation index maps were generated using 80% of the trial, while source reconstruction was applied to all trials.

#### 2.4.5 Digit-maps

To further characterize neural activity associated with different finger movement across broad temporal scales and fine spatial resolution using MEG, we attempted to map finger movement activation onto the sensorimotor cortex through classification techniques. The analysis focused on frequency bands which demonstrated the ability to effectively differentiate between finger movements (see Section 3.4).

The source-reconstructed signals within ROI were classified for the four tasks at each vertex using a 50 ms time window. For each vertex, both the four-class classification accuracy and the accuracies for the individual classes were recorded. At the individual level, task preference for each vertex was determined by comparing the classification accuracies among the four classes. Each vertex was labeled according to the class with the highest accuracy, resulting in a time-resolved digit map for each participant.

At the group level, vertices showing mean four-class accuracies significantly above the 25% chance level were first identified to form the vertex cluster in each window. Within identified clusters, the number of participants whose vertices were labeled as each of the four classes was counted, and the class with the largest number of participants was assigned as the group label for that vertex. In this way, a group-level digit map with 50-ms temporal resolution was obtained through a voting-based procedure.

### 2.5 Statistics

Nonparametric Friedman tests, which are suitable for comparing repeated measures across multiple conditions, were used to evaluate differences in (1) reaction times and movement times across tasks (Section 2.4.1), (2) accuracies derived from single versus combined time-window features (Section 2.4.4). Post hoc pairwise comparisons were conducted using two-sided Wilcoxon signed-rank tests, and the resulting *p*-values were corrected for multiple comparisons using the Benjamini–Hochberg procedure (Benjamini & Hochberg, 1995). Wilcoxon signed-rank tests were also used to assess differences between classification accuracies and the 25% chance level across different conditions in Sections 2.4.1 and 2.4.4.

Aligned-Rank Transform Analysis of Variance (ART-ANOVA) (Wobbrock et al., 2011) was applied to evaluate classification accuracies across spatial feature extraction methods and six frequency bands (Section 2.4.4). ART-ANOVA is a nonparametric factorial approach that enables testing of main and interactions effects in multi-factor and repeated-measures designs. Post hoc pairwise comparisons were performed using estimated marginal means (EMMs) derived from the ART linear model, with *p*-values adjusted using Tukey’s method.

Cluster-based nonparametric permutation tests (5,000 permutations) were employed to assess differences between task and baseline periods in the TFR analyses (Section 2.4.3), and to evaluate deviations of window-wise and point-wise classification accuracies from chance level (Section 2.4.4). This approach emphasized the temporal or frequency continuity of significant effects. Additionally, one-sided non-parametric permutation tests with Benjamini–Hochberg correction were employed to evaluate vertex-wise classification accuracies against chance within the digit maps (Section 2.4.5).

## 3 Results

### 3.1 EMG-based assessment of motor execution and task discriminability

**Fig. 2.**
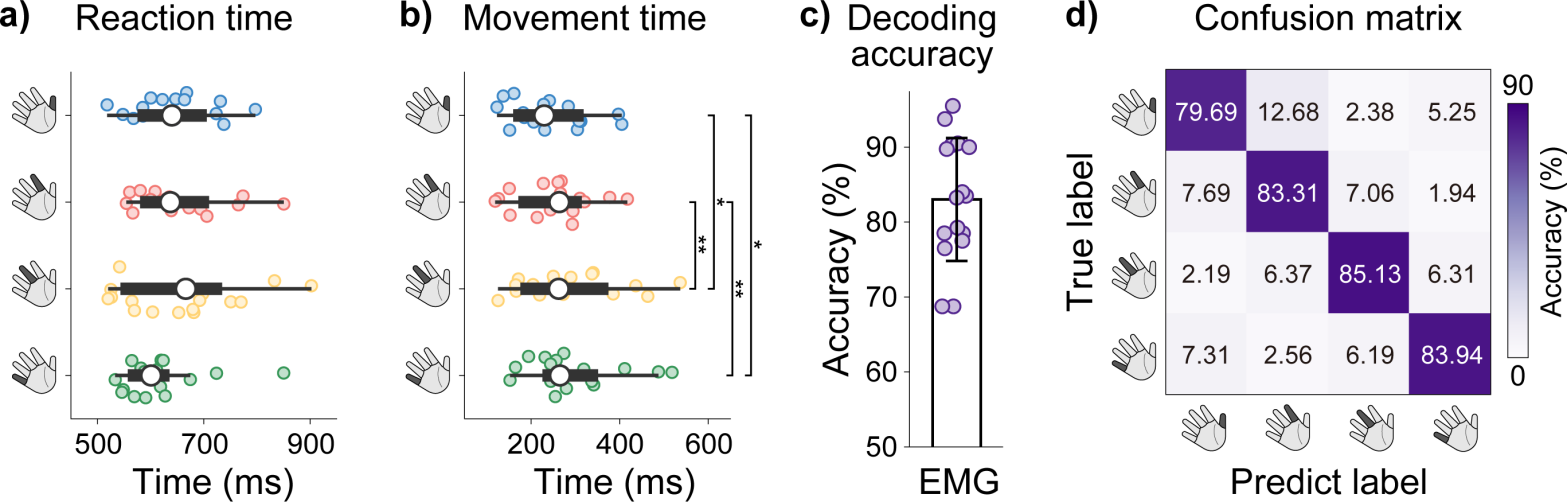
Task execution parameters and EMG-based decoding results. (a) Group-level reaction times for the four tasks. (b) Group-level movement times for the four tasks. In (a) and (b), the left and right edges of each box represent the 1st quartile (Q1, 25th percentile) and the 3rd quartile (Q3, 75th percentile), respectively, with the central marker indicating the median. Whiskers extend to [Q1−1.5 × IQR, Q3 + 1.5 × IQR], where IQR denotes the interquartile range. Statistical significance was evaluated using the Friedman test, followed by post hoc pairwise comparisons performed with Wilcoxon signed-rank tests and Benjamini–Hochberg correction. *p < 0.05; **p < 0.01. (c) Four-class classification accuracy based on EMG signals. (d) Confusion matrix for four-class classification, aggregated across all participants and validation folds.

Figure 2a and b shows the group-level reaction times and movement times across the four tasks. Participant-level results are provided in Supplementary Tables S1 and S2. The average reaction time across all 16 participants was 642.65 ± 89.74 ms, with no significant differences among tasks (Friedman test, χ^2^(3) = 4.2750, *p* = 0.2333, n = 16). The average movement times were 241.18 ± 87.10 ms, 252.72 ± 85.96 ms, 288.60 ± 119.34 ms, and 294.50 ± 104.94 ms for the four tasks, respectively, and significant differences were observed (Friedman test, χ^2^(3) = 27.8250, *p* < 0.001, n = 16). Post hoc Wilcoxon signed-rank tests with Benjamini–Hochberg correction revealed significant differences between the Thumb task and the Middle (*Z* = -3.4128, *p* = 0.0019) and Little tasks (*Z* = -3.5162, *p* = 0.0019), as well as between the Index task and Middle (*Z* = -2.7923, *p* = 0.0105) and Little tasks (*Z* = -2.6371, *p* = 0.0125), while no other pairwise comparisons were significant (see Supplementary Table S3 for details). Figure 2c and d displays the EMG classification results and the corresponding four-class confusion matrix across participants. Participant-level accuracies are provided in Supplementary Table S4. The group-average classification accuracy was 83.02% ± 8.20%, significantly above the 25% chance level (Wilcoxon signed-rank tests, *Z* = -3.517, *p* = 0.0004). Together, these results indicate that the four finger extension tasks were performed consistently across trials, and that EMG patterns elicited by different movements exhibited strong discriminability, providing a solid basis for subsequent decoding of task-related cortical neural signals.

### 3.2 Characteristics of different frequency band signals during movement execution

**Fig. 3.**
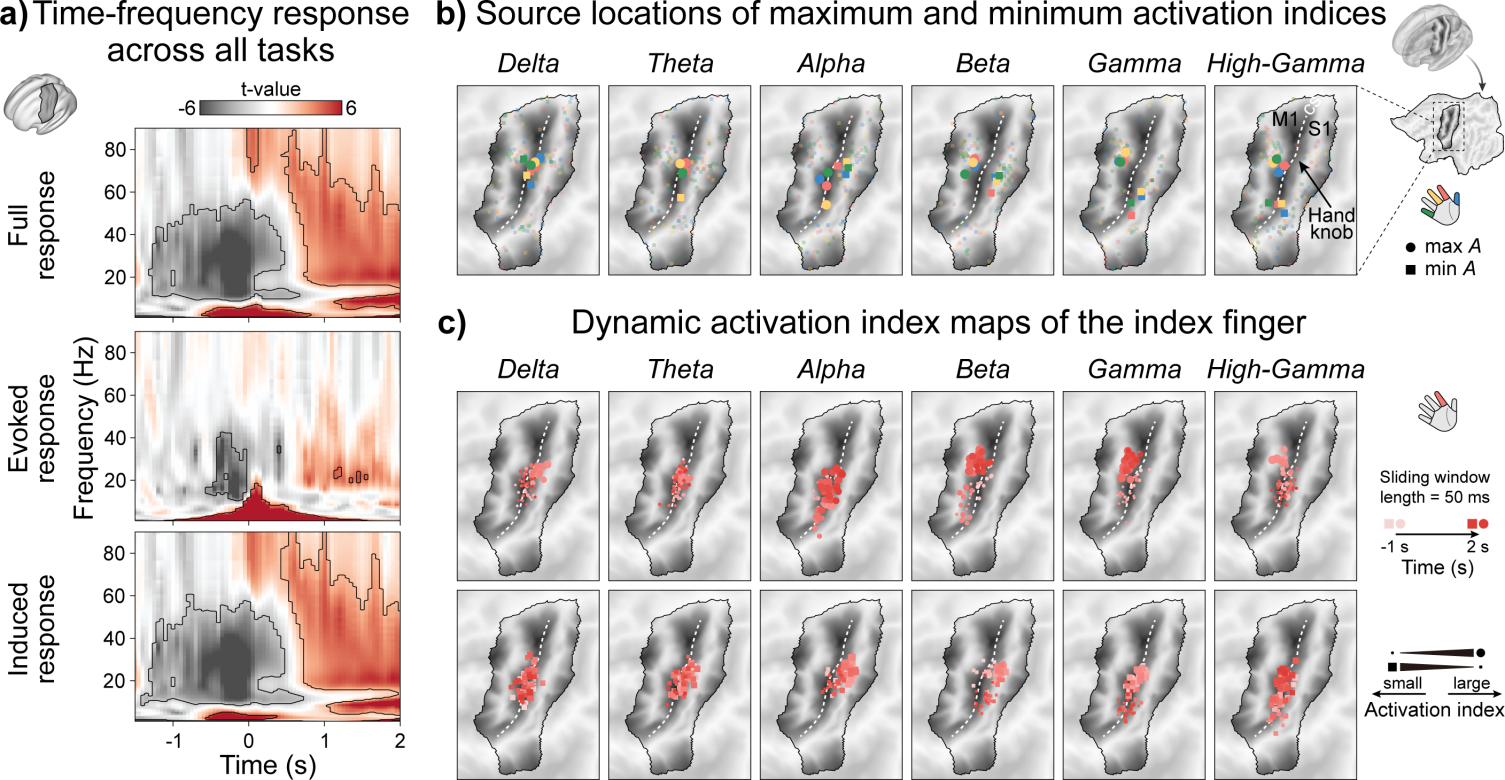
Group-averaged MEG source signals characteristics reflecting movement-related neural activity. (a) Task-averaged time–frequency responses (TFRs) averaged across all vertices within the region-of-interest (ROI). TFRs are displayed as *t*-values obtained from statistical comparison between the task and baseline periods. Black solid lines indicate statistically significant regions (cluster-based permutation test, 5,000 permutations, *p* < 0.05). (b) Locations of maximum and minimum activation indices in the full-epoch activation index (*A*-epoch) maps across all frequency bands and tasks. Large opaque markers denote group-level averaged locations, whereas smaller, more transparent markers indicate individual participant results. (c) Locations of maximum (top row) and minimum (bottom row) activation indices in the short-window activation index (*A*-window) maps for the Index task across all frequency bands. Marker color represents time, and marker size reflects normalized *A* magnitude.

Group-averaged TFRs across all tasks are shown in the top row of Figure 3a, representing the mean activity of all vertices within the ROI. In the δ and θ bands, clear power increases were observed during task execution. The δ-band enhancement began before movement onset and persisted beyond it, lasting longer than the movement time, consistent with recent findings (Noel et al., 2025). The θ-band activation followed a similar pattern but was shorter in duration. In the α and β bands, marked power decrease occurred from the preparation phase through execution, followed by strong power rebound after movement termination. The γ-band showed similar power change patterns to a lesser extent. Notably, task-related power decrease extended continuously from the α to the γ range, while power increase exhibited a clear boundary around 13 Hz. The high-γ-band exhibited a significant power increase both during movement execution and after movement offset.

The TFR results described above were obtained by averaging the TFRs computed from single trials. In addition, we recalculated the TFRs using the trial-averaged signals, which are referred to as evoked responses in previous studies (Gohil et al., 2024). The evoked response represents the phase-locked components shared across trials, whereas the induced response reflects non–phase-locked components and was derived by subtracting the evoked response from the full TFR. The new results are shown in the middle and bottom rows of Figure 3a. As illustrated, the δ- and θ-band exhibited more sustained evoked responses, lasting longer than those observed in the full TFR. Notably, in the α-band, we observed a pronounced evoked power increase around 0 s, a feature that has received limited emphasis in prior movement-related studies. In addition, the α, β, and γ bands showed evoked power decreases before movement onset, and the β-band also displayed a brief evoked increase after movement termination. In contrast, no clear evoked responses were observed in the high-γ-band. The TFR results for all four tasks are shown in Supplementary Figure S1.

Figure 3b shows the locations of the maximum and minimum activation indices in the *A*-epoch maps across all frequency bands and four tasks. Circular and square markers indicate maximum and minimum locations, respectively. These locations were also used to extract spatial features for the *A*-epoch-based classification (Section 2.4.4). Although the locations of maxima and minima were spatially dispersed across tasks and frequency bands, no distinct or consistent task-specific spatial separation was observed. Notably, in the α, β and γ bands, the maximum-*A* and minimum-*A* locations tended to separate along the anterior–posterior direction of the central sulcus, with maxima located closer to the precentral gyrus and minima to the postcentral gyrus. Both task-averaged *A*-epoch maps and *A*-window maps are presented in Supplementary Figure S2.

Figure 3c shows the distributions of the maximum-*A* (top row) and minimum-*A* (bottom row) locations of the *A*-window maps across all frequency bands, using the Index task as an example. The color of each marker represents time, while its size corresponds to the normalized *A* magnitude. The results for all four tasks are shown in Supplementary Figure S3.

In the δ and θ bands, the size of the maximum-*A* location markers increased and then decreased over time, reflecting a temporal evolution of *A* magnitude similar to the power dynamics observed in the TFR. These maximum-*A* locations progressively clustered around the hand-knob region, reaching their greatest concentration near 0 s when the activation indices peaked. Afterward, they became more dispersed. This pattern indicates that δ- and θ-band activations are closely related to finger movement execution. In contrast, the minimum-*A* locations exhibited neither substantial size changes nor clear spatial clustering over time. In the α-band, the minimum-*A* locations were more consistently clustered across time windows than the maximum-*A* locations, mainly distributed on the posterior side of the central sulcus (i.e., the postcentral gyrus). In the β and γ bands, the maximum-*A* and minimum-*A* locations showed a clearer separation between the precentral and postcentral gyri. The clustering of maximum-*A* locations became more prominent over time, corresponding to the power increase observed in the TFR results, whereas the minimum locations clustered mainly during the middle of the task period, consistent with the power decrease. In the high-γ-band, the maximum locations were concentrated in the precentral gyrus around 0 s, while the minimum locations showed no evident temporal trend, similar to those in the δ and θ bands.

These results indicate that the δ and θ bands exhibited task-related activations during movement execution, with their strongest activations clustered around the hand-knob region. The α, β, and γ bands showed ERD beginning before movement onset and continuing through the execution phase, followed by ERS after movement termination. The cortical distributions of these ERS and ERD patterns were largely segregated along the anterior and posterior banks of the central sulcus. Among them, the α-band showed a more complex pattern, characterized by an evoked response around movement onset. The high-γ-band displayed a slight power increase during execution, with activation centered in the precentral gyrus. However, cortical activation patterns did not show clear spatial segregation across different finger movements.

### 3.3 The impact of spatiotemporal representations different frequency band signals on task decoding

**Fig. 4.**
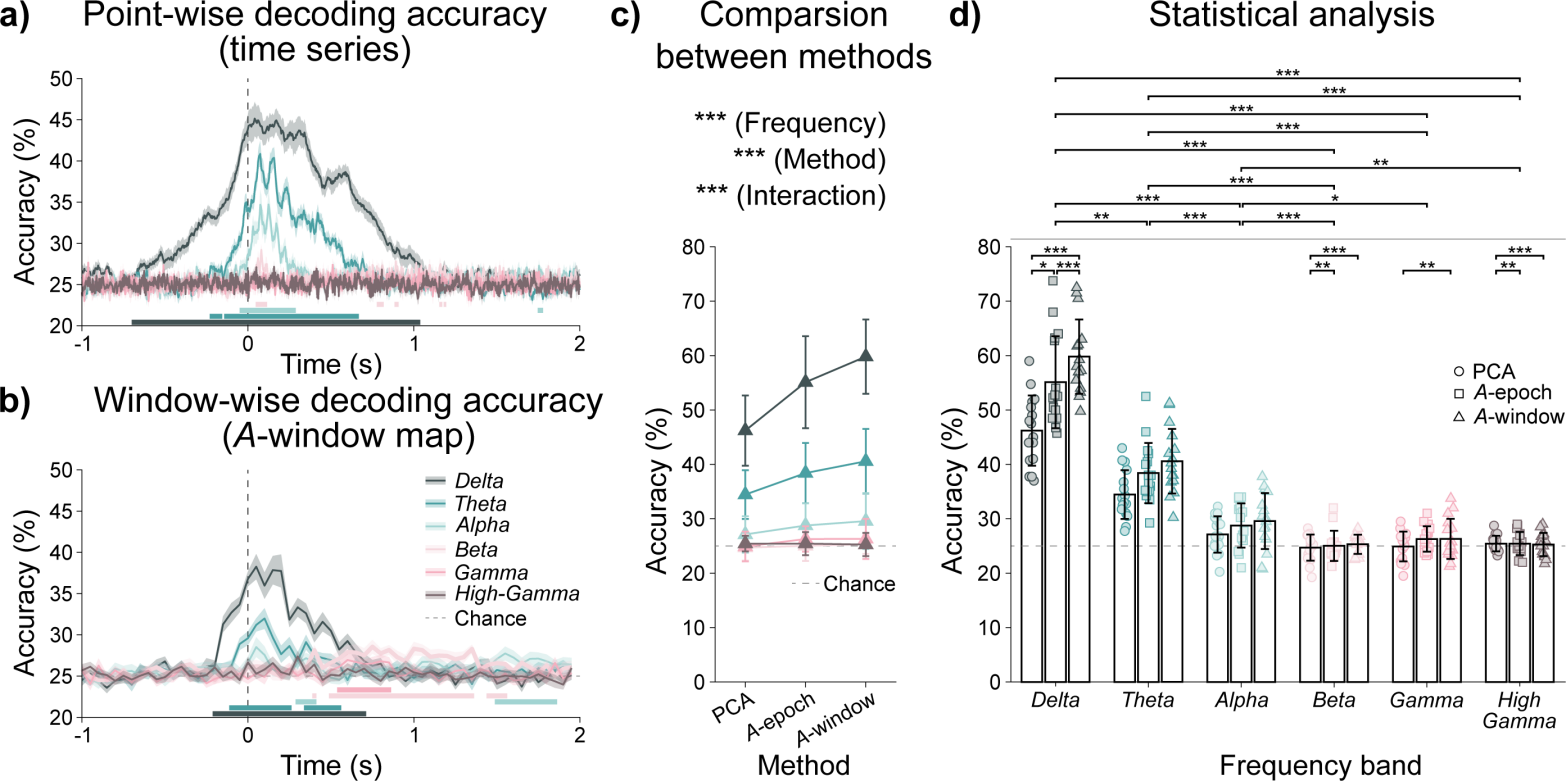
Classification accuracy results. (a) Source-reconstructed signals point-wise classification results across all frequency bands. (b) *A*-window maps window-wise classification results across all frequency bands. In (a) and (b), gray lines indicate the chance level of 25%. Shaded bands indicate the standard error of the mean (SEM) across participants. And the bars under accuracy plots indicate periods significantly higher than the chance level (cluster-based permutation test, 5,000 permutations, *p* < 0.05). (c) Classification accuracies obtained using three spatial features extraction methods across six frequency bands (Two-way ART-ANOVA, frequency: ***p < 0.0001, method: ***p < 0.0001, interaction: ***p < 0.0001). (d) Statistical comparisons of accuracies using a two-way ART-ANOVA with Tukey’s adjustment for post hoc multiple comparisons. The gray dashed line indicates the chance level of 25%. The error bars denote the standard deviation of accuracies. *p < 0.05, **p < 0.01, ***p < 0.001.

Figure 4a shows the group-averaged point-wise classification results across frequency bands using reconstructed signals from all vertices within the ROI. Cluster-based permutation tests identified time intervals of significantly above-chance accuracy in the δ, θ, and α bands, indicating periods during which the four tasks could be reliably distinguished. In the δ-band, effective decoding extended over -698.75 ms–1037.92 ms, with a peak accuracy of 45.13% at 41.25 ms. The θ-band showed a shorter effective decoding duration of -228.75 ms–667.92 ms, with peak accuracy reaching 40.85% at 74.58 ms. The α-band exhibited limited task-related discriminability within the 48.75 ms–287.92 ms interval, with lower overall accuracy and a maximum of 34.72%. In the β-band, brief above-chance performance was observed during movement execution and in the ERS period after 500 ms. By contrast, decoding in the γ and high-γ bands remained at chance level across the entire epoch.

Figure 4b shows the group-averaged window-wise classification accuracies using *A*-window maps for each frequency band. Only the δ- and θ-band activation index maps exhibited clear discriminability around the movement-related activation period. The δ-band showed significant above-chance accuracy from –200 to 750 ms, lasting longer than the θ-band (–100–300 ms and 350–600 ms). For the other bands, with the exception of high-γ-band, overall accuracies remained close to chance level, yet cluster-based permutation tests identified significant deviations from chance during certain intervals. In the β and γ bands, significant intervals were observed mainly after 500 ms (β: 500–1400 ms and 1450–1600 ms; γ: 550–900 ms), that is, following movement termination, which may suggest that spatial information associated with ERS in these bands differed across tasks. In the α-band, significant intervals were found at 300–450 ms and 1500–1900 ms. Although the high-γ-band showed task-related spatial patterns, it did not yield discriminability.

To further evaluate the contribution of spatial information to decoding performance, we compared four-class classification results obtained from three types of spatial features: PCA components, spatial locations derived from *A*-epoch maps, and spatial locations derived from *A*-window maps across multiple time windows. Participant-level results are provided in Supplementary Tables S5–S7. As shown in Figure 4c, classification accuracies in the δ, θ, and α bands remained consistently and significantly above chance (Wilcoxon signed-rank tests with Benjamini–Hochberg correction, see Supplementary Table S8 for details).

To evaluate the effects of the three spatial feature extraction methods and six frequency bands on decoding performance, an ART-ANOVA was conducted with method and frequency band as factors. The analysis revealed significant main effects of both factors, as well as a significant interaction between them. Post hoc comparisons showed that decoding accuracy in the δ-band (PCA-based: 46.22% ± 6.46%; *A*-epoch-based: 55.11% ± 8.47%; *A*-window-based: 59.81% ± 6.83%) was significantly higher than in all other bands. The θ-band achieved the second highest accuracy (PCA-based: 34.45% ± 4.48%; *A*-epoch-based: 38.41% ± 5.53%; *A*-window-based: 40.58% ± 5.94%) and significantly outperformed the α-band (PCA-based: 27.13% ± 3.34%; *A*-epoch-based: 28.77% ± 4.07%; *A*-window-based: 29.58% ± 5.14%). In contrast, the β, γ, and high-γ bands yielded lower accuracies than the first three bands, with no significant differences among them. Across all frequency bands, the *A*-window-based method achieved the highest overall accuracy, whereas the PCA-based method performed the weakest.

Given the significant interaction between factors, additional post hoc analyses were conducted for the three methods within each frequency band (Fig. 4d). In the δ-band, classification accuracy consistently increased from PCA-based method to *A*-window-based method. No significant differences among methods were observed in the θ and α bands. Although PCA-based method accuracies tended to exceed those of *A*-based methods in the β, γ, and high-γ bands, these accuracies did not differ significantly from the chance level. This apparent reversal of the approach effect likely reflects statistical variability rather than a meaningful difference in decoding performance. Full statistical results are reported in Supplementary Tables S9–S11.

Overall, the results demonstrate that task-related neural activity in the δ, θ, and α bands consistently supported discrimination of single-trial finger movements. Among these, the δ-band provided the highest classification performance and the longest discriminative duration, spanning the preparation, execution, and post-movement periods, indicating that it carries movement-specific information throughout the task. The θ-band yielded intermediate performance, while both θ and α bands conveyed most of their discriminative information around the movement execution phase. Moreover, spatial activation patterns in the contralateral sensorimotor cortex, particularly in the δ and θ bands, offered strong discriminability among individual finger movements. In contrast, although β, γ, and high-γ bands exhibited task-related activity patterns, they did not reliably distinguish between finger movements under the present decoding framework. Finally, enhancing the spatiotemporal resolution of these features further improved decoding accuracy, underscoring the critical contribution of spatial information to single-trial finger movement decoding.

### 3.4 Impact of spatiotemporal information expansion on decoding capability

In Section 3.3, which examined the influence of spatial information on decoding performance, two main observations emerged. First, the discriminability provided by task-related spatial activation patterns varied over time. Second, even when spatial information within the ROI was spatially blurred and not distinctly different across tasks (PCA-based method), the corresponding temporal signals still supported successful task discrimination. These results indicate that the time-resolved spatial activation patterns and frequency-specific amplitude modulations each provides distinct and informative contributions for distinguishing finger movements. To more comprehensively capture this information, we extracted the spatial locations from all above-chance time windows in the δ and θ bands (Fig. 4b) and used the corresponding time series segments (Fig. 4a) for subsequent decoding. To reduce feature dimensionality, the δ- and θ-band time series were downsampled to 30 Hz prior to segmentation extraction. The α to high-γ bands were excluded due to low decoding performance (Fig. 4a, b) and the absence of significant differences among spatial feature extraction approaches (Fig. 4c, d).

**Fig. 5.**
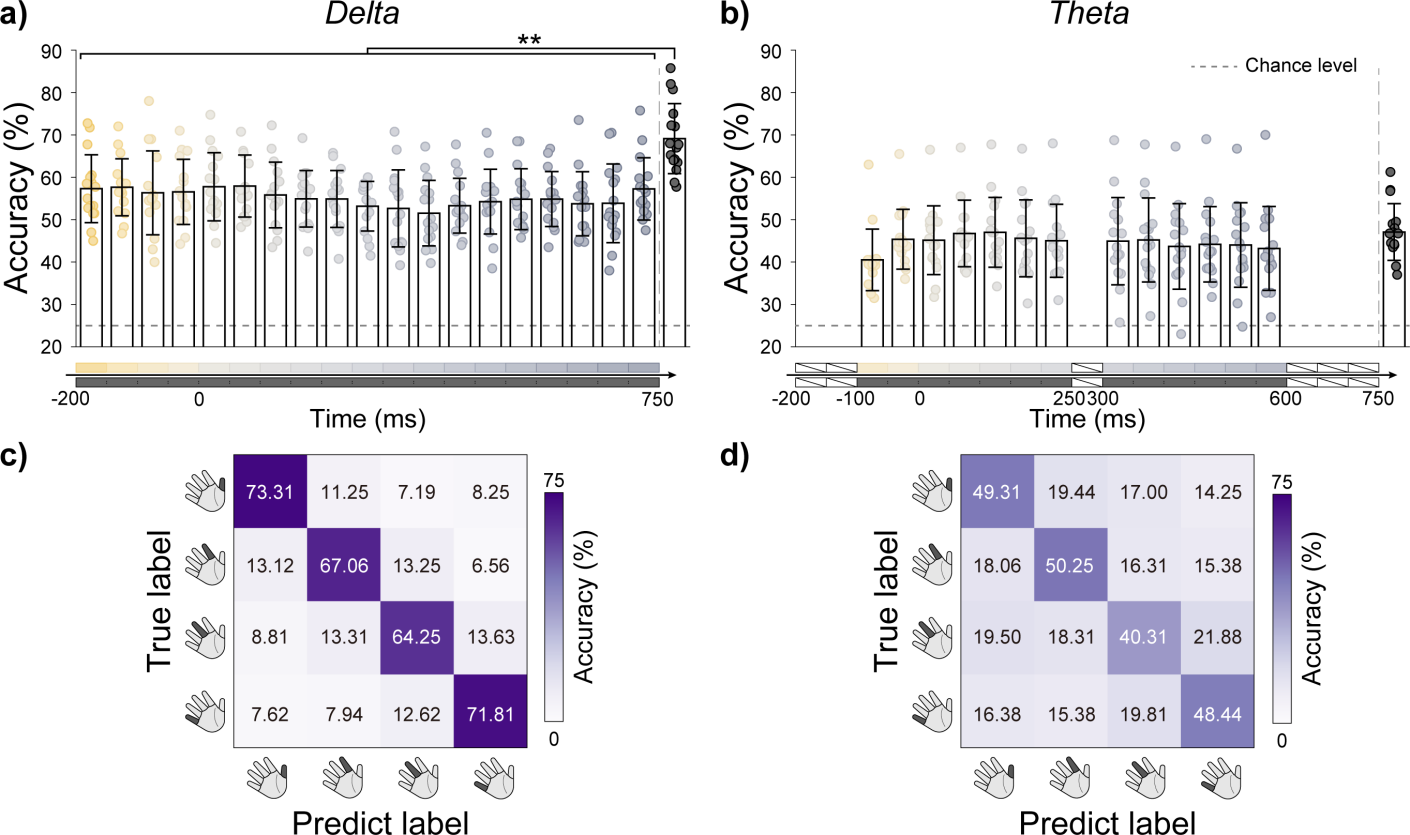
Classification results with extended spatiotemporal features in the δ and θ bands. (a) Classification accuracy based on features extracted from individual time windows and their combination in the δ-band. **p < 0.01. (b) Classification accuracy based on features extracted from individual time windows and their combination in the θ-band. In the (a) and (b), black dash lines indicate the chance level. (c) Confusion matrix for four-class classification based on δ-band features, aggregated across all participants and validation folds. (d) Confusion matrix for four-class classification based on θ-band features, aggregated across all participants and validation folds.

We compared classification accuracies using spatial features from individual decodable windows versus the combination of all such windows. Results are presented in Figure 5a and b, with accuracy details provided in Supplementary Tables S12 and S13. In the δ-band, the highest decoding accuracy was achieved by combining information across 19 windows (69.11% ± 8.27%). A Friedman test indicated significant differences among feature sets (*χ²*(19) = 93.827, *p* < 0.001, n = 16), and post hoc Wilcoxon signed-rank tests with Benjamini–Hochberg correction confirmed that this combining window approach yielded significantly higher accuracy than any single-window condition (see Supplementary Table S14 for details). These results indicate that combining spatial and temporal feature diversity enhances decoding performance.

By contrast, this effect was not observed in the θ-band. Although classification accuracies varied across different windows (Friedman test: χ²(13) = 22.551, *p* < 0.05, n = 16), post hoc Wilcoxon tests with Benjamini–Hochberg correction revealed no significant differences between multi-window and single-window features (see Supplementary Table S15 for details). This discrepancy may reflect differences in the information carried by the two bands and underscores the broader temporal discriminative capacity of the δ-band for finger movements.

In addition, confusion matrices were computed for both the δ- and θ-band classifications using features from all windows (Fig. 5c, d). In the δ-band, higher classification accuracies were observed for the thumb and little finger, whereas distinguishing between index and middle finger movements was more difficult. Misclassifications tended to occur between neighboring fingers. The θ-band exhibited a broadly similar pattern, although overall confusion levels were higher and the adjacency effect was less pronounced. In this case, the index task emerged as the least confusable, while the middle task remained the most difficult to classify across both frequency bands. Notably, this pattern contrasts with the EMG-based classification results, in which the middle task showed the highest discriminability. This discrepancy indicates that the task-relevant features contributing to decoding differ between muscle activity and cortical neural signals.

### 3.5 Cortical characterizations of neural activity associated with finger movements

Finger movement is a dynamic process involving sustained temporal engagement and widespread cortical neural activity. A few discrete vertices on the cortex cannot fully capture this complexity, as the underlying neural mechanisms extend across both temporal and spatial dimensions. For better assessing this complexity, the analysis was extended to cover a broader region of the contralateral sensorimotor cortex and a longer time span.

**Fig. 6.**
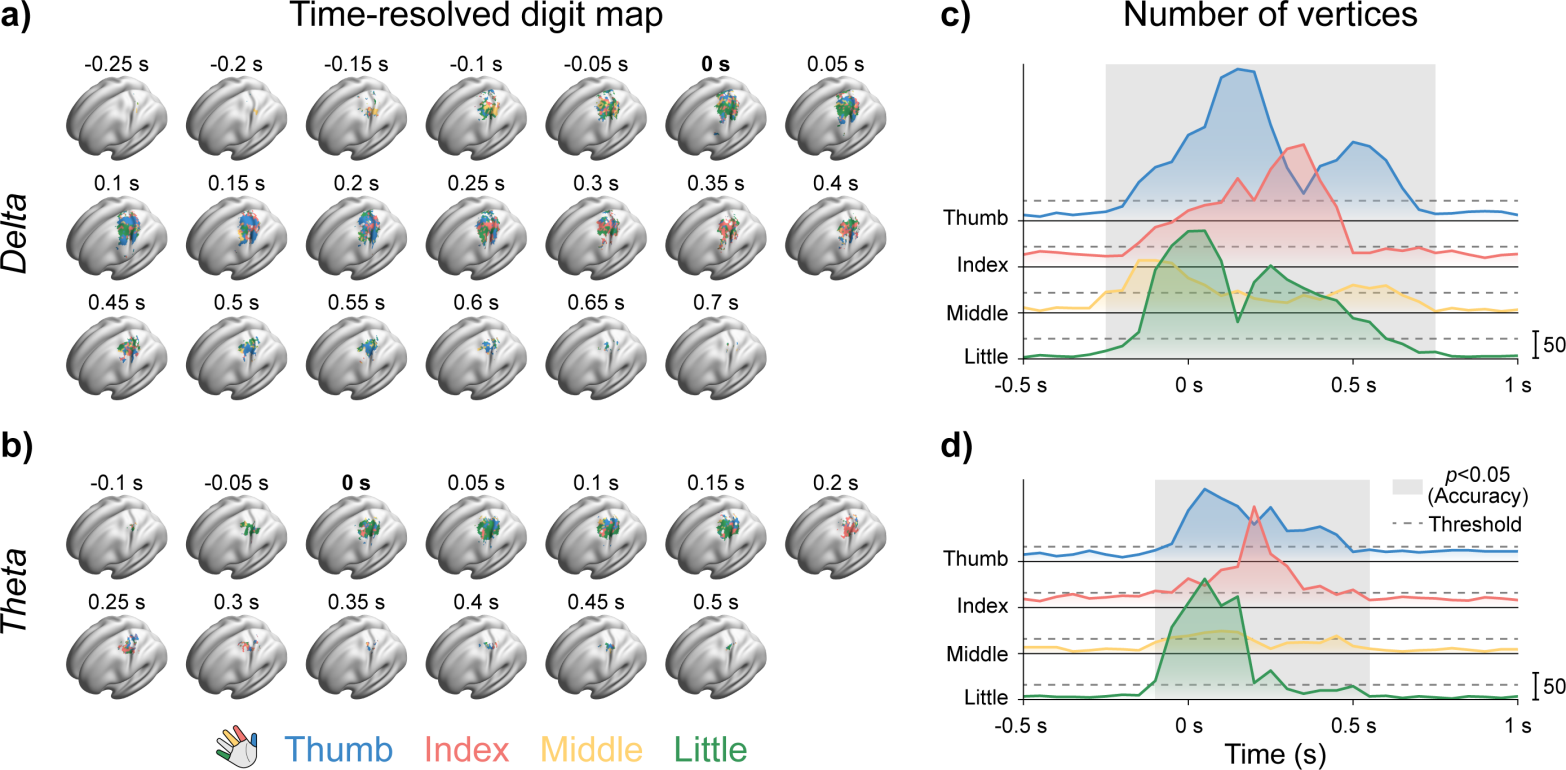
Cortical neural activity maps for four finger movements with 50 ms temporal resolution. (a) Group-level digit maps in the δ-band. (b) Group-level digit maps in the θ-band. (c) Number of vertices assigned to each finger in the group-level δ-band digit maps. (d) Number of vertices assigned to each finger in the group-level θ-band digit maps. In (c) and (d), black dashed lines denote the activation threshold, defined as 25% of one-fourth of the maximum total vertex count across all time windows. Gray rectangles indicate the corresponding time periods represented in (a) and (b).

We generated digit maps for the δ and θ bands (Fig. 6a, b), which had previously been shown to discriminate the four tasks, as shown in Section 3.3. Colored vertices indicate grid points where four-class classification accuracy was significantly above the 25% chance level, as determined by 5,000 permutation tests with Benjamini–Hochberg correction. For the α-band, we also attempted to construct digit maps; however, the results did not survive statistical testing. In these digit maps, each vertex within a given time window was assigned to the finger associated with the highest classification accuracy. Group-level digit maps were then obtained by applying a voting procedure at each vertex across participants. This yielded non-overlapping clusters of vertices preferentially associated with one of the four fingers, effectively mapping their cortical representations in each time window. Across time, the four finger maps displayed a progressive trend toward greater spatiotemporal separability.

Additionally, to characterize the temporal dynamics of cortical activations underlying the four finger movements, we quantified the number of vertices assigned to each finger in the group-level digit maps across time windows (Fig. 6c, d). For both frequency bands, we summed the number of vertices assigned to each finger across time windows and set a threshold at 25% of one-fourth of the maximum total, indicated by black dashed lines in the figure.

In the δ-band, finger-specific activations occurred between –250 and 700 ms, closely matching the decoding duration in the window-wise classification result (Fig. 4b). Among the four fingers, the middle finger map activated first but showed the shortest duration, the fewest vertices, and thus the smallest cortical representation. The little finger map reached its peak area near movement onset (0 ms) and consistently contained more vertices than the middle finger throughout the activation period. The thumb map became dominant around 100 ms and exhibited the longest activation duration (–250 to 700 ms). The index finger map emerged latest, around 300 ms, with a shorter activation period (–250 to 500 ms) but the largest total number of vertices across all windows.

A similar temporal sequence was observed in the θ-band, with the little finger, thumb, and index finger successively becoming dominant. However, the activation periods for all fingers were shorter than those in the δ-band. The middle finger map again showed the shortest activation duration, but unlike in the δ-band, it never became dominant at any time.

## 4 Discussion

In this study, we combined MEG with source imaging to capture the dynamic representations of contralateral sensorimotor activity associated with individual finger movements. We then evaluated the decoding capacity of these signals from three complementary perspectives: frequency, space, and time. Our results demonstrate that δ- and θ-band activity can reliably discriminate among the four finger movements, with the δ-band achieving the highest decoding accuracy of 69.11% ± 8.27%. Spatial activation patterns from these bands alone were sufficient for classification, and refining spatial resolution further improved decoding performance. Incorporating the temporal dynamics of spatial information yielded additional gains in accuracy. Together, these findings highlight the critical role of sub-8 Hz neural activity in representing individual finger movements and underscore the potential of MEG, with its high spatial and temporal resolution, for advancing research in motor neurophysiology and neural decoding.

### 4.1 Neural signals below 8 Hz may contribute significantly to the decoding of limb movements

Our results demonstrate that neural activity below 8 Hz (δ and θ bands) enables the most effective decoding of individual finger movements, consistent with many previous studies (Bu et al., 2023; Quandt et al., 2012; Sun et al., 2024). The superior decoding capacity of sub-8 Hz activity is not limited to differentiating hand movements. Prior work have shown that neural dynamics in this frequency range can also decode upper and lower limb movements (Wang et al., 2024), as well as kinematic information, such as limb position and movement speed (Iturrate et al., 2018; Jerbi et al., 2011). Taken together, these findings suggest that sub-8 Hz neural activity carries critical motor control information within the primary sensorimotor cortex and plays a pivotal role in decoding.

However, the differences observed between the δ and θ bands suggest that they may serve distinct functions during movement. δ-band activity showed the strongest discriminability among finger movements, with activation and decodable periods (Fig. 4a, b) beginning before movement onset and extending beyond the termination of EMG activity. This indicates that it may encode information beyond mere muscle activity. Moreover, combining features from multiple δ-band time windows significantly improved classification accuracy, suggesting that the information carried by this band evolves dynamically over time.

These findings imply that δ-band activity may convey continuously updated information related to movement intention, limb control, and the functional state of sensorimotor cortex. Similar findings have been reported in previous studies. For example, Noel et al. demonstrated that the emergence of movement intention is accompanied by increased δ-band activation in the motor cortex (Noel et al., 2025). In addition, Hamel-Thibault et al. (2018) further showed that δ-band activity can predict action selection prior to movement onset. In addition, δ oscillations have been proposed to organize the temporal dynamics of sensorimotor processing and encode temporal contextual information for sensory inputs (Morillon et al., 2019). Our findings may provide further evidence supporting these perspectives.

For the θ-band, both the activation period and the decodable window were more closely aligned with the duration of movement (Fig. 4a, b), suggesting a stronger coupling with muscle activity. This observation is consistent with the findings of Huang et al.(2024) who combined EMG and MEG recordings. Our results further indicate that θ-band activity does not reliably differentiate between individual finger movements, and the information it conveys at different time points appears to be less distinctive. These findings suggest that θ-band primarily reflects the activation state of the sensorimotor cortex during motor execution.

The specific roles of δ and θ activity in motor control, however, remain incompletely understood and warrant further investigation in future studies.

### 4.2 Classification performance of α, β, γ, and high-γ bands

In our results, α-band cortical activity was able to distinguish the four tasks, but with limited accuracy. This suggests that the α-band may carry only a modest amount of task-relevant information for finger motor control. Notably, as shown in Figure 3, the α-band exhibited ERD patterns similar to those of the β-band in the induced response. However, the two bands demonstrated clearer distinctions in the timing and frequency characteristics of their ERS. In addition, a pronounced power increase in α-band was observed around movement onset in the evoked response. These findings suggest that α-band activity may involve more complex functional processes during motor control. Further work is needed to clarify the specific role of α-band dynamics in movement execution. In contrast, although the β, γ, and high-γ bands signals exhibited clear task-related features, they did not yield superior decoding performance in this study.

These findings contrast with some previous reports (Lee et al., 2022; Zubarev et al., 2024), particularly regarding the α and β bands. We propose several possible explanations for this discrepancy:

1. Weakness and variability of neural oscillations across single trials likely reduced their discriminative power in this study. This variability may arise from two main sources. First, detecting movement onset using EMG introduces slight temporal jitter, which can lead to phase misalignment of neural oscillations across trials and result in the loss of consistent features—an effect that becomes more pronounced at higher frequencies. Second, the inherently low signal-to-noise ratio of single-trial recordings may hinder the detection of stable and clearly distinguishable neural activity. Importantly, this trial-to-trial instability can potentially be mitigated. Huang et al. (2024) proposed a source imaging approach that projects MEG data based on EMG activity, demonstrating its ability to reliably extract frequency-specific neural signals in the primary motor cortex associated with finger movement. Such approaches should be considered in future studies to improve the consistency and discriminability of neural features.
2. Feature extraction and classification methods employed in this study may not have been optimally sensitive to neural oscillatory changes, potentially limiting the extraction of stable information. We used only time-domain signals as inputs for classification, whereas previous studies have suggested that power-based features may yield better decoding performance. To examine this possibility, we performed a simple validation analysis. Specifically, for all six frequency bands, classification was repeated using both PCA-based and *A*-epoch map-based methods, but replaced the time series inputs by TFR of the reconstructed signals. Detailed methods and results are provided in Supplementary Analysis S1, Supplementary Analysis Figure 1, and Supplementary Analysis Tables 1–5. The results showed that, when using spatial features derived from the *A*-epoch maps, classification accuracies in the α (28.39% ± 3.74%), β (30.09% ± 2.05%), γ (27.58% ± 3.46%), and high-γ (27.02% ± 2.93%) bands were significantly above chance. Although accuracies in the δ (PCA-based: 33.22% ± 5.40%; *A*-epoch map-based: 38.38% ± 7.54%) and θ (PCA-based: 27.63% ± 4.04%; *A*-epoch map-based: 34.13% ± 4.84%) bands were lower than those obtained with time-domain features, they remained substantially higher than those of the other four bands, further confirming the decoding advantage of δ and θ activity. These findings demonstrate the feasibility of decoding based on power dynamics. Furthermore, the use of more advanced feature extraction techniques and classifiers, such as Convolutional Neural Network (CNN) (Tao et al., 2022; Zubarev et al., 2024) or EEG-Net (Ding et al., 2025), holds considerable potential for further improving classification performance.
3. Neural oscillations in the α, β, γ and high-γ bands may primarily represent the active states of neuronal populations involved in limb motor control, rather than encoding fine details of movement execution. Cheyne et al. reviewed MEG studies of oscillatory activity in human sensorimotor cortex, suggesting that these oscillations contribute to tracking movement-related parameters, such as hand position and muscle length (Cheyne, 2013). Some decoding studies have successfully classified movements based on oscillatory features by distinguishing between contralateral and ipsilateral limb movements, leveraging differences in activation states of the left and right sensorimotor cortices (Pfurtscheller et al., 2006). Moreover, oscillatory activity has been used to decode continuous movements or gestures (Lee et al., 2022; Zubarev et al., 2024), where sustained movement states provide stable information for classification. Recent work on rhythmic finger-movement decoding using broadband cortical signals further showed that α- and β-band activity can effectively decode continuous repetitive actions, whereas lower-frequency components contributed less (Ding et al., 2025). In contrast, our study focused on single, brief finger movements with shorter durations, in which oscillatory features may carry less discriminative information. This interpretation is consistent with a recent finger-movement study, which reported that low-frequency features better differentiated finger identity, whereas α/β-band activity was more strongly related to movement state (Sun et al., 2024). Nonetheless, the present study cannot further dissociate these oscillatory mechanisms. Future work will be needed to determine how these bands differentially represent continuous and single movement events.

### 4.3 MEG can capture the representation of finger movements on the sensorimotor cortex

MEG has previously been reported to precisely capture spatial patterns of neural activity associated with finger movements. Huang et al. (2024) demonstrated that δ-, θ-, and β-band MEG signals can accurately localize finger movement-related activations within the primary motor cortex. In the present study, we further show that MEG, with its high temporal and spatial resolution, effectively captures the dynamic neural processes underlying finger movements within the contralateral sensorimotor cortex. Moreover, extracting and leveraging spatial information through source imaging technique played a key role to improving classification accuracy. In addition, source imaging likely contributed to effective decoding by improving signal quality through noise suppression. In previous studies that performed four-finger decoding directly at the sensor level, a classification accuracy of 57% was achieved (Quandt et al., 2012). In comparison, our approach reached an accuracy of 69.11% ± 8.27%, which may be attributed to the enhanced spatial information extraction and noise reduction afforded by source reconstruction.

Furthermore, our results demonstrate that MEG can capture distinct spatial activation patterns of the δ and θ bands across different finger movements. Building on this, digit maps of δ and θ bands were generated to characterize cortical biases associated with finger motor control. Additionally, we also observed that α-, β-, γ-, and high-γ-band activity exhibited a consistent spatial trend in which ERD activity was primarily localized to the postcentral gyrus, while ERS activity was concentrated in the precentral gyrus. This spatial dissociation may reflect functional differences in oscillatory processes during movement and highlights the ability of MEG to characterize the spatiotemporal organization of sensorimotor activity.

However, our characterization of movement-related cortical activation patterns still has limitations. First, the activation patterns associated with the four finger movements showed substantial overlap, without clearly segregated spatial clusters. Similar findings have been reported in functional magnetic resonance imaging (fMRI) and invasive recording studies (Ejaz et al., 2015; Jorge et al., 2020), which describe finger representations as distributed rather than discretely localized. Second, the spatial extent of activation revealed by MEG appeared broader than that commonly observed in fMRI (Huber et al., 2020). While differences in spatial resolution may explain this discrepancy, it is also important to note that MEG captures neural activity directly and with millisecond temporal precision. Recent work has reported co-active regions involved in movement control (Gordon et al., 2023). The broad activation observed in our study may, therefore, encompass additional movement-related neural activity, which could be challenging to detect with lower temporal resolution.

Overall, our findings suggest that finger movement representations are not confined to spatially segregated activation clusters, but instead emerge as dynamic and distributed activation patterns across the contralateral primary sensorimotor cortex. These results underscore the value of MEG in characterizing the temporal evolution of cortical activity during fine motor control. Future work should further refine MEG-based spatiotemporal decoding approaches to better leverage these dynamic neural representations.

### 4.4 Effect of muscle activation patterns on cortical decoding

In this study, EMG signals were used to assess participants’ motor performance and were further subjected to four-class classification. As evidenced by the differences in movement times across tasks and the strong classification performance achieved with only two EMG channels (Section 3.1), muscle activation patterns exhibited high task discriminability, surpassing that of cortical signals.

An important question is the extent to which finger-specific muscle activation patterns contribute to decoding performance. Although each finger has characteristic movement and muscle activation profiles that might be expected to influence MEG-based decoding, our analyses suggest that these differences are not strongly reflected in the cortical features used for classification. The following two results support this interpretation. First, although δ-, θ-, and α-band MEG signals supported four-class discrimination, their decoding performance and informative periods differed markedly. Notably, δ-band activity remained decodable far beyond the movement time. This extended discriminability indicates that δ-band cortical activity captures motor-related information beyond muscle activation, highlighting its unique contribution to decoding. Second, the EMG- and MEG-based confusion matrices showed distinct task-specific patterns. For example, the thumb was hardest to classify using EMG but among the easiest using MEG, whereas the middle finger showed the opposite trend. This divergence indicates that the two modalities rely on different task-relevant features, supporting the view that cortical decoding is not simply a reflection of muscle activation patterns.

The enslaving effect, referring to the involuntary co-movement of non-target fingers during single-finger actions (Zatsiorsky et al., 2000), may also have influenced cortical decoding performance in this study. Although individuals can achieve independent control of each finger, unintentional co-activation commonly occurs during natural motor behavior (Noort et al., 2016; Schneider, 2020). The underlying mechanisms and cortical representations of this effect remain unclear, leaving its impact on decoding accuracy uncertain. To reduce this potential influence, the ring finger, which is most difficult to move independently, was excluded from the experiment. Participants were instructed to generate the intention to move only the indicated finger and to perform the most isolated extension possible. Nevertheless, the limited number of EMG channels prevented detailed recording of finger-specific muscle activity or motion trajectories. Consequently, we were unable to quantify co-movements or fully evaluate their effects on cortical representations and decoding outcomes.

Understanding how muscle activity patterns are represented in cortical signals is crucial for clarifying the neural mechanisms of finger movement. Future studies should apply higher-resolution EMG and motion-tracking techniques to investigate these relationships in greater detail.

### 4.5 Cortical processing from visual Input to motor output

In visually guided motor decoding studies, the influence of visually evoked responses on decoding performance has long been a topic of discussion. For example, Bu et al. (2023) reported that including MEG channels covering the occipital cortex improved classification accuracy in a gesture decoding task, highlighting the critical role of occipital regions in guiding action execution and representing action concepts.

In the present study, we employed source imaging to isolate task-related neural activity originating from the contralateral primary sensorimotor cortex, thereby minimizing the contribution of signals from other brain regions, including visual cortices. To further examine the potential influence of visual responses on decoding finger movements under our experimental design, we conducted an additional analysis. Specifically, PCA components explaining 70% of the variance were computed for each of the 68 cortical parcellations, and multivariate pattern analysis (MVPA) was performed from 500 ms before cue onset onward to characterize the temporal evolution of task-related responses across different cortical areas. Detailed methods and results are provided in Supplementary Analysis S2 and Supplementary Analysis Figure 2.

The supplementary analysis revealed a clear temporal sequence of cortical involvement during task processing. The bilateral lateral occipital cortices were the first regions to achieve above-chance decoding accuracy, emerging approximately 100 ms after cue onset. Decoding peaks then progressively shifted anteriorly to the parietal cortex, which also exhibited the longest sustained decoding period. Meanwhile, decoding accuracy in the left precentral and postcentral gyri gradually increased and reached its peak around 600 ms, closely corresponding to the group-averaged reaction time. In contrast, decoding accuracy in the right sensorimotor cortex remained relatively low throughout the epoch.

These spatiotemporal dynamics likely reflect the sequential neural processes underlying visually guided motor execution. The lateral occipital cortex, as the primary recipient of visual input, generated early responses corresponding to task-specific cues. The parietal cortex, often regarded as an integrative hub for sensory–motor information—including visual, spatial, and proprioceptive inputs—then exhibited task-related activity, suggesting its involvement in early movement planning, spatial attention, and body-state representation (Fogassi & Luppino, 2005; Freedman & Ibos, 2018; Whitlock, 2017). Finally, task-specific differentiation emerged in the primary sensorimotor cortex, corresponding to the generation of motor output. The observed hemispheric asymmetry may further reflect the unilateral nature of the task. While occipital activity was largely bilateral and non-lateralized, left-hemispheric dominance in the parietal and sensorimotor areas likely resulted from the right-hand movement execution.

These findings suggest that while visual responses are engaged at the initial stages of task processing, their influence on decoding performance is minimal, underscoring the effectiveness of isolating sensorimotor signals through source reconstruction.

### 4.6 Limitation and future work

First, our decoding framework relied on a limited number of vertex-level features and single-band representations within the contralateral sensorimotor cortex, thereby capturing only a portion of the underlying spatiotemporal structure of movement-related cortical activity. Future work should, therefore, focus on developing more effective feature extraction strategies that can systematically integrate spatial, temporal, and spectral information across frequency bands. Furthermore, incorporating activity from additional motor-related regions such as the ipsilateral sensorimotor cortex, premotor cortex, and supplementary motor area, may provide complementary discriminative information and further enhance classification performance. It should be acknowledged that the beamforming approach employed in this study assumes independence among neural sources (Van Veen et al., 1997), whereas activity within these motor-related regions is often highly correlated. Future work could address this limitation by adopting enhanced formulations, such as multi-source minimum variance beamformers (Moiseev et al., 2011) or adaptive beamformer based on sparse Bayesian algorithm (Cai et al., 2023), which are better suited for reconstructing multiple correlated generators. Alternatively, minimum-norm–based reconstruction methods, including weighted minimum norm estimate (wMNE) and standardized low-resolution electromagnetic tomography (sLORETA), may provide more stable estimates when extended or correlated activation patterns are expected (Feng et al., 2025). In addition, cross-frequency coupling between neural oscillations may encode richer information related to motor intention or execution, which warrants further investigation.

Second, more advanced feature extraction and classification methods should be considered to further improve accuracy. Recent studies have demonstrated the efficacy of deep learning techniques in feature extraction and classification of EEG and MEG signals (Bu et al., 2023; Ding et al., 2025; Tao et al., 2022; Zubarev et al., 2024), which we aim to investigate in future work. We believe that deep learning models hold significant potential for analyzing high-dimensional data from multiple virtual channels at the source level, offering a promising direction for advancing movement decoding accuracy.

The SQUID-MEG system has inherent technical limitations due to its fixed sensor array, which cannot adapt to different head sizes and may result in subtle head movements during recording. Another technical constraint is the attenuation of magnetic signals with increasing distance between cortical sources and sensors, which can compromise decoding accuracy. Addressing these issues requires stabilizing the sensor–head relative position and minimizing scalp-to-sensor distance.

A promising solution lies in wearable optically pumped magnetometer (OPM)-MEG system. Future work will focus on utilizing this wearable system, in anticipation that it will significantly enhance decoding performance. Their portability and flexible sensor placement can overcome many of the above limitations and are expected to significantly enhance decoding performance. Beyond improving signal quality, OPM-MEG also holds strong potential for BCI applications. While our study remains far from achieving a fully operational BCI with real-time feedback, it demonstrates the feasibility of MEG as a powerful neural measurement tool for finger movement decoding. The wearability of the OPM system will further support their potential for widespread use in BCI research, as shown in recent studies utilizing steady-state visual evoked potentials (Wittevrongel et al., 2021). Future work will explore OPM-based decoding of finger movements and motor imagery, aiming to advance BCI technologies and applications.

## 5 Conclusion

In this study, we characterized neural representation associated with finger extension movements using MEG and performed decoding by integrating features across temporal, frequency, and spatial dimensions. Our findings demonstrate that neural activity below 8 Hz, particularly in the δ-band, provides a strong cortical representation of the differences among unilateral finger movements, yielding a peak average decoding accuracy of 69.11% ± 8.27%. These distinctions were reflected not only in the spatial organization of cortical activation patterns but also in the extended temporal evolution of these signals, which persisted well beyond the duration of muscle activity. In addition, the results highlight the strength of MEG combined with source imaging in capturing the fast-evolving spatiotemporal dynamics underlying movement-related cortical representations. These results could potentially increase our understanding of the neural mechanisms underlying limb movement control in the sensorimotor cortex, and provide valuable insights into the potential applications of MEG and low-frequency neural signals for BCI development.

## Supplementary Material

Supplementary Material

## Data Availability

The group-level data files to generate the figures are available at https://doi.org/10.6084/m9.figshare.28623506. The raw MEG and MRI data cannot be available according to the ethical permission. The custom code used for the analysis pipeline and results visualization is available at https://github.com/Yzheng9725/MEG-study-fingermovement.
