# Supplementary material for: Multidimensional dynamic characterization and decoding of finger movements using magnetoencephalography
Source: Imaging Neurosci (Camb). 2026 Mar 30;4:IMAG.a.1182. doi: 10.1162/IMAG.a.1182 (PMC13037661; doi:10.1162/IMAG.a.1182)
Supplement: Supplementary Material [file IMAG.a.1182_supp.pdf]

Supplementary Analysis 1

To evaluate whether power dynamics can provide effective decoding, we performed the following analyses for signals in all six frequency bands.

First, we extracted two types of features following the procedures described in Section 2.4.4: (1) principal component analysis (PCA) components explaining 70% of the variance within the contralateral sensorimotor cortex, and (2) source-reconstructed signals from the strongest task-related activation locations identified in the full-epoch activation index maps. For each feature type, full time–frequency responses (TFRs) were computed using Morlet wavelet analysis, with four cycles and an output temporal resolution of 50 ms. The frequency resolution was set to 0.5 Hz below 8 Hz and 1 Hz between 8 and 90 Hz.

Four-class classification was then performed using a linear support vector machine (SVM) with a one-versus-rest (OVR) strategy, consistent with the approach in Section 2.4.4. Statistical significance of classification accuracies relative to the 25% chance level was assessed using Wilcoxon signed-rank tests with Benjamini-Hochberg correction. Differences in classification accuracy obtained using different spatial feature extraction methods across six frequency bands were evaluated using Aligned-Rank Transform Analysis of Variance (ART-ANOVA), followed by post-hoc comparisons performed using estimated marginal means, with *p*-values adjusted using Tukey's method.

Group-averaged classification results are presented in Supplementary Analysis Figure 1, while participant-level results and detailed statistical outcomes are provided in Supplementary Analysis Tables 1-5.

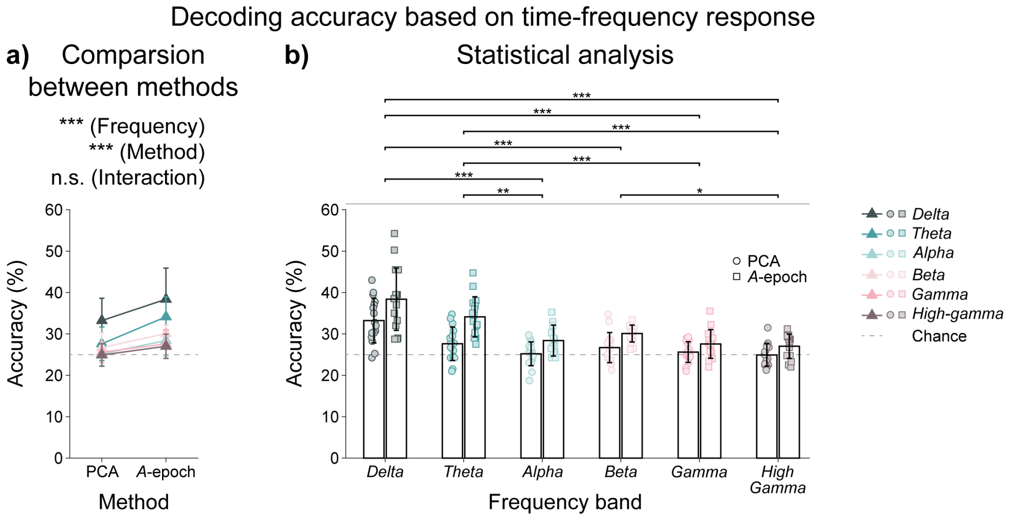

Supplementary Analysis Figure 1. Classification results with time-frequency responses. a) Classification accuracies obtained using two spatial features extraction methods across six frequency bands (Two-way ART-ANOVA, frequency: ***$p < 0.0001$, method: ***$p < 0.0001$, interaction: $p = 0.0929$). **b)**

Statistical comparisons of accuracies using a one-way ART-ANOVA with Tukey's adjustment for post hoc multiple comparisons. The gray dashed line indicates the chance level of 25%. The error bars denote the standard deviation of accuracies. *$p < 0.05$, **$p < 0.01$, ***$p < 0.001$.

Supplementary Analysis Table 1. PCA-based classification accuracy using TFR across all frequency bands.

Participant	*δ*	*θ*	*α*	*β*	*γ*	high-*γ*
P01	24.25%	24.25%	25.50%	24.25%	24.75%	22.50%
P02	39.50%	27.75%	28.75%	27.50%	27.75%	23.25%
P03	37.75%	30.75%	23.75%	23.75%	24.25%	27.75%
P04	30.75%	27.75%	24.75%	22.75%	25.25%	22.50%
P05	30.25%	32.50%	28.00%	27.25%	23.00%	27.25%
P06	32.75%	26.00%	20.75%	28.50%	28.50%	31.50%
P07	37.00%	21.50%	23.00%	28.75%	24.75%	22.00%
P08	40.00%	34.75%	26.75%	28.50%	25.25%	21.25%
P09	28.25%	28.00%	18.75%	34.75%	29.00%	25.75%
P10	25.25%	24.50%	25.75%	22.75%	26.25%	24.00%
P11	36.25%	21.00%	26.25%	33.00%	25.75%	23.50%
P12	29.25%	29.00%	29.75%	27.75%	21.75%	25.00%
P13	32.00%	24.25%	24.75%	21.25%	24.50%	26.75%
P14	43.00%	33.75%	27.00%	24.50%	28.50%	25.75%
P15	30.25%	26.75%	23.00%	25.25%	21.00%	22.75%
P16	35.00%	29.50%	26.75%	26.50%	29.25%	27.00%
Mean	33.22%	27.63%	25.20%	26.69%	25.59%	24.91%
SD	5.40%	4.04%	2.88%	3.65%	2.51%	2.71%

Supplementary Analysis Table 2. Classification accuracy using TFR with spatial locations derived from full-epoch AI maps across all frequency bands.

Participant	δ	θ	α	β	γ	high-γ
P01	35.00%	37.50%	24.25%	32.00%	27.75%	29.75%
P02	45.50%	34.50%	33.00%	27.50%	35.50%	22.50%
P03	39.50%	38.00%	26.00%	26.50%	24.50%	28.75%
P04	29.00%	32.00%	25.00%	31.25%	24.00%	22.00%
P05	37.75%	36.75%	25.25%	33.50%	30.25%	28.50%
P06	28.75%	29.00%	27.00%	29.00%	28.50%	24.75%
P07	39.50%	29.00%	25.50%	30.00%	32.25%	28.00%
P08	32.00%	36.00%	27.00%	31.50%	26.75%	28.75%
P09	28.75%	33.00%	35.25%	31.75%	28.50%	29.25%
P10	33.25%	27.50%	28.75%	29.50%	25.50%	24.75%
P11	39.50%	29.00%	32.00%	29.75%	25.75%	29.75%
P12	38.50%	35.00%	26.50%	32.00%	30.00%	24.75%
P13	45.50%	32.25%	24.25%	31.25%	29.00%	23.25%
P14	50.25%	44.75%	30.25%	29.25%	22.00%	29.50%
P15	54.25%	41.50%	35.25%	26.25%	23.50%	31.25%
P16	37.00%	30.25%	29.00%	30.50%	27.50%	26.75%
Mean	38.38%	34.13%	28.39%	30.09%	27.58%	27.02%
SD	7.54%	4.84%	3.74%	2.05%	3.46%	2.93%

Supplementary Analysis Table 3. Post-hoc pairwise comparisons between classification accuracies across frequency bands and chance-level using Wilcoxon signed-rank tests.

	Z	*p*
δ - PCA	3.4133	0.0019
δ - *A*-epoch	3.5197	0.0017
θ - PCA	2.1987	0.0418
θ - *A*-epoch	3.5185	0.0017
α - PCA	0.4918	0.6283
α - *A*-epoch	/	0.0026
β - PCA	1.6305	0.1373
β - *A*-epoch	3.5174	0.0017
γ - PCA	0.8810	0.4540
γ - *A*-epoch	2.5087	0.0242
high-γ - PCA	/	0.6283
high-γ - *A*-epoch	2.2782	0.0389

Supplementary Analysis Table 4. Post-hoc pairwise comparisons of classification accuracies across different frequency bands.

Contrast	estimate	SE	df	t.ratio	*p*
δ vs θ	32.34	11.7	75	2.760	0.0755
δ vs α	81.62	11.7	75	6.966	<0.0001
δ vs β	57.03	11.7	75	4.867	0.0001
δ vs γ	84.88	11.7	75	7.243	<0.0001
δ vs high-γ	93.81	11.7	75	8.006	<0.0001
θ vs α	49.28	11.7	75	4.206	0.0010
θ vs β	24.69	11.7	75	2.107	0.2951
θ vs γ	52.53	11.7	75	4.483	0.0004
θ vs high-γ	61.47	11.7	75	5.246	<0.0001
α vs β	-24.59	11.7	75	-2.099	0.2992
α vs γ	3.25	11.7	75	0.277	0.9998
α vs high-γ	12.19	11.7	75	1.040	0.9028
β vs γ	27.84	11.7	75	2.376	0.1780
β vs high-γ	36.78	11.7	75	3.139	0.0282
γ vs high-γ	8.94	11.7	75	0.763	0.9729

Supplementary Analysis Table 5. Post-hoc pairwise comparisons of classification accuracies with different methods.

Contrast	estimate	SE	df	t.ratio	*p*
PCA vs *A*-epoch	-49	7.17	15	-6.837	<0.0001

Supplementary Analysis 2

To investigate how different cortical regions respond during the process from visual cue presentation to motor task execution, we performed pointwise decoding across time for source-reconstructed signals from all brain parcellations within a Multivariate pattern analysis (MVPA) framework. MEG signals band-pass filtered between 0.5 and 90 Hz were first projected onto 68 cortical parcellations across the whole brain, and epochs were defined from 500 ms before to 2000 ms after the onset of the visual cue. Principal component analysis (PCA) was then applied separately to the reconstructed signals within each parcellation, and components explaining 70% of the variance were retained.

Subsequently, four-class decoding was performed point by point over time using the PCA components from each parcellation as input features. The analysis was implemented using the MVPA-Light toolbox with a SVM classifier and five-fold cross-validation. Supplementary Analysis Figure 2(a) shows the results for key regions, including the bilateral lateral occipital cortex, bilateral superior and inferior parietal cortices, and bilateral precentral and postcentral gyri, while Supplementary Analysis Figure 2(b) presents the temporal evolution of classification accuracy across all 68 parcellations in 50 ms steps throughout the entire epoch.

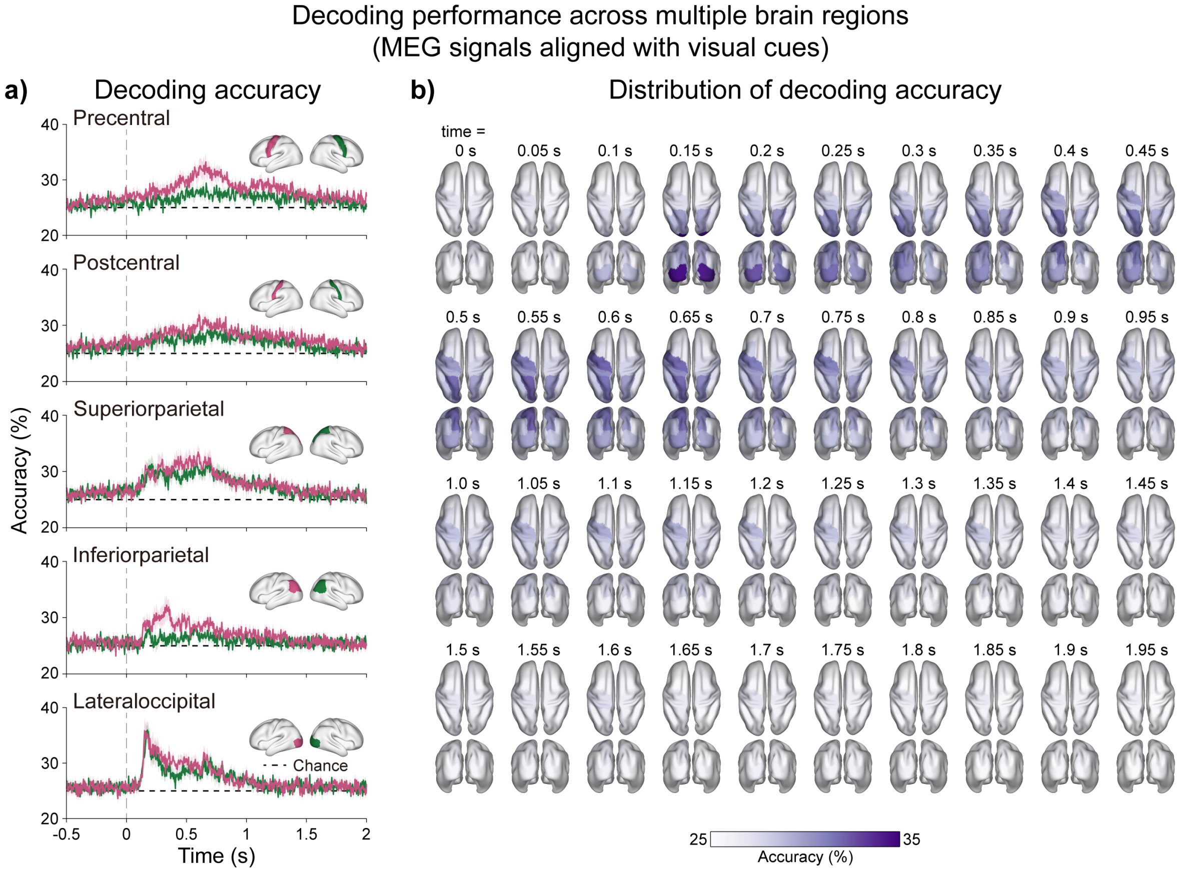

Supplementary Analysis Figure 2. Group-averaged four-class classification results. a) Group-

averaged classification accuracies for the bilateral lateral occipital cortex, bilateral superior and inferior parietal cortices, and bilateral precentral and postcentral gyri. Time 0 indicates the onset of the visual cue. Results from the left hemisphere are shown in pink, and those from the right hemisphere are shown in green. The gray dashed line indicates the chance level of 25%. And shaded bands indicate the SEM across participants. **b)** Temporal evolution of group-averaged classification accuracy across all 68 cortical parcellations. Classification accuracies are shown in 50 ms steps, with time 0 indicating the onset of the visual cue.

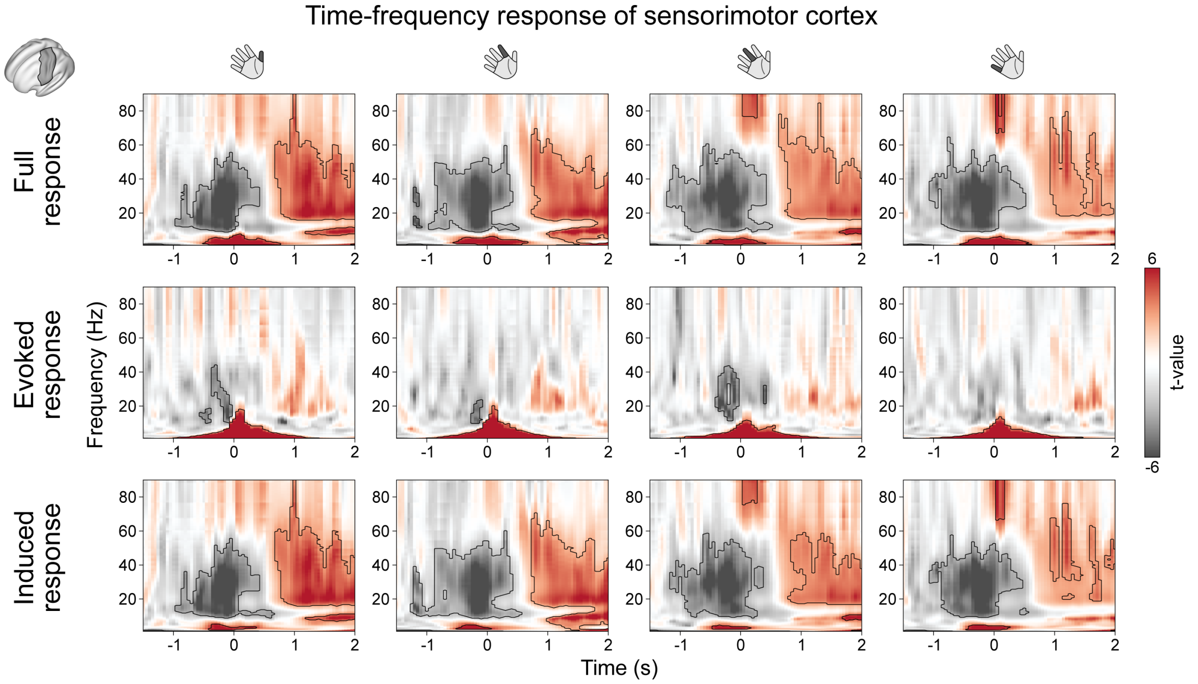

Supplementary Figure 1. Group-averaged time-frequency responses (TFRs) within the left precentral and postcentral gyri across the four tasks. TFRs are represented as *t*-values maps, reflecting group-level deviations from baseline, averaged across all vertices within the region-of-interest (ROI). Black solid lines indicate statistically significant regions (cluster-based permutation test, 5000 permutations, $p < 0.05$). *Top row*: Group-averaged full TFRs. *Middle row*: Group-averaged evoked responses. *Bottom row*: Group-averaged induced responses.

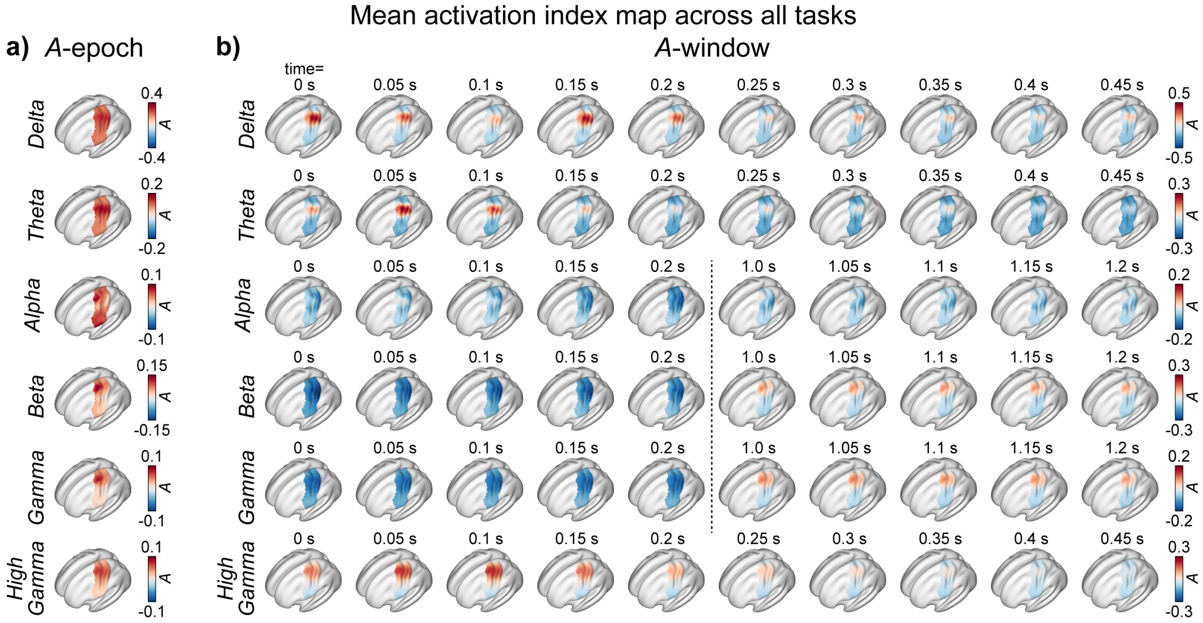

Supplementary Figure 2. Task-averaged full-epoch activation index (*A*-epoch) maps and short-window activation index (*A*-window) maps across all frequency bands.

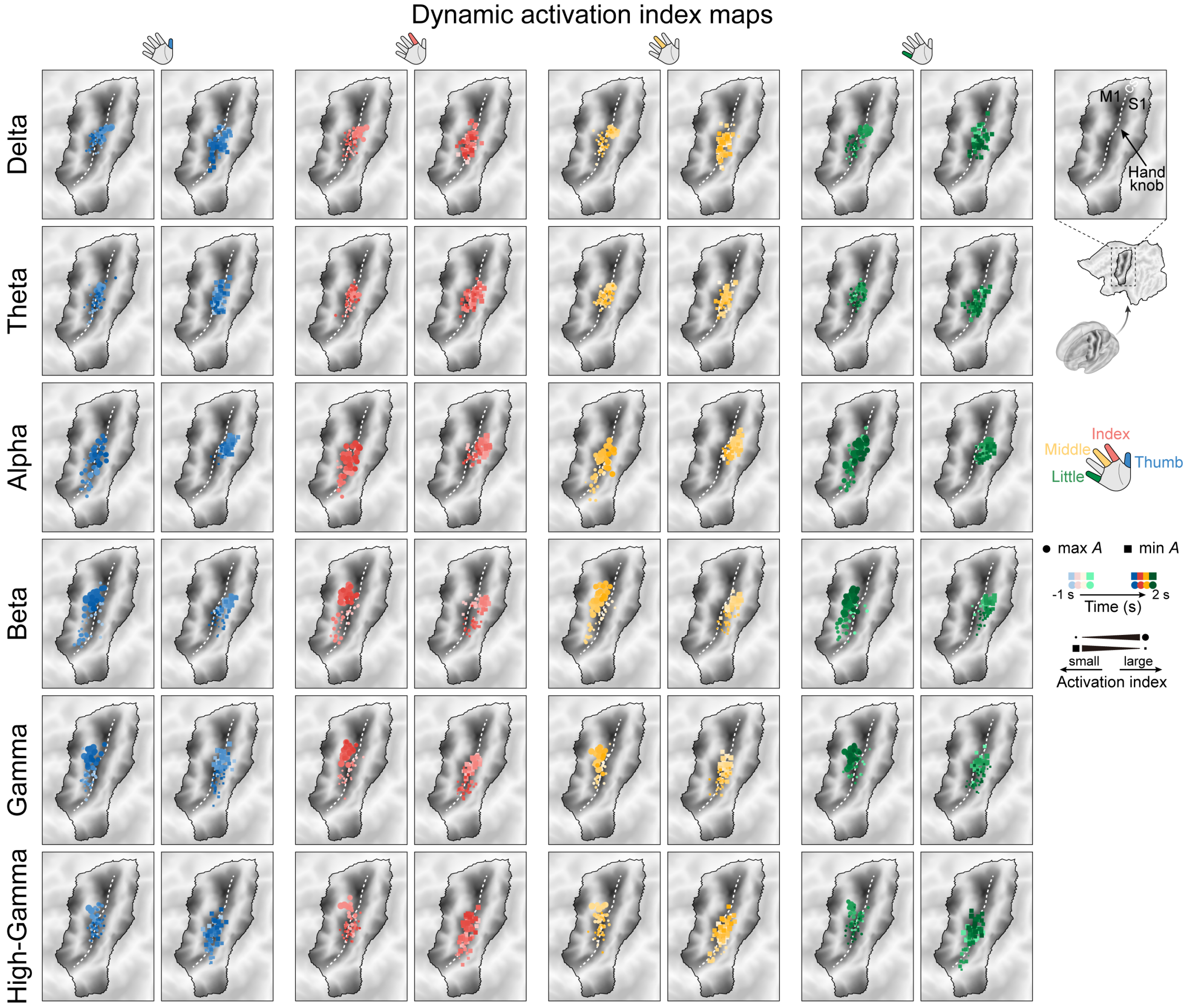

Supplementary Figure 3. Locations of maximum and minimum activation indices in the short-window activation index maps for all tasks. Marker color represents time, and marker size reflects normalized *A* magnitude.

Supplementary Table 1. Reaction times across four tasks.

Participant	Thumb (ms)	Index (ms)	Middle (ms)	Little (ms)
P01	585.60 ± 97.57	566.40 ± 94.68	603.64 ± 93.07	569.31 ± 103.63
P02	585.08 ± 76.88	560.59 ± 76.30	541.59 ± 90.92	533.35 ± 76.20
P03	667.64 ± 159.59	694.16 ± 152.99	832.92 ± 180.46	674.61 ± 150.63
P04	644.09 ± 116.24	608.32 ± 110.95	564.77 ± 91.99	611.08 ± 118.98
P05	647.06 ± 95.62	641.12 ± 128.49	750.84 ± 138.44	619.68 ± 105.88
P06	622.14 ± 138.17	672.14 ± 145.16	697.68 ± 146.97	618.51 ± 70.50
P07	600.40 ± 72.11	635.14 ± 96.07	525.25 ± 70.10	548.48 ± 75.99
P08	635.65 ± 129.01	580.45 ± 90.27	569.44 ± 86.73	581.85 ± 114.81
P09	731.17 ± 110.35	705.69 ± 103.47	692.17 ± 90.62	564.55 ± 93.39
P10	547.80 ± 95.94	599.89 ± 129.55	526.15 ± 124.40	627.30 ± 139.57
P11	518.04 ± 76.86	554.32 ± 108.12	520.05 ± 98.97	545.91 ± 106.53
P12	723.20 ± 127.68	764.97 ± 182.72	769.96 ± 126.30	623.54 ± 102.15
P13	663.66 ± 100.26	638.69 ± 95.51	652.77 ± 105.45	590.60 ± 100.83
P14	567.63 ± 82.83	629.12 ± 93.41	81.11 ± 110.81	588.33 ± 79.97
P15	737.66 ± 92.28	773.64 ± 114.67	79.39 ± 79.06	723.79 ± 99.80
P16	796.90 ± 126.48	850.34 ± 139.93	902.28 ± 132.05	850.19 ± 136.33
Mean	642.11 ± 76.25	654.69 ± 84.82	656.88 ± 116.12	616.94 ± 79.09

Supplementary Table 2. Movement durations across four tasks.

Participant	Thumb (ms)	Index (ms)	Middle (ms)	Little (ms)
P01	206.03 ± 50.78	227.79 ± 62.64	252.55 ± 53.07	273.92 ± 55.88
P02	241.42 ± 40.75	266.26 ± 42.52	289.97 ± 56.45	279.76 ± 53.79
P03	404.81 ± 87.85	417.42 ± 70.95	463.47 ± 114.40	518.21 ± 129.84
P04	397.19 ± 68.83	377.53 ± 90.47	536.60 ± 131.42	487.62 ± 88.32
P05	151.75 ± 32.07	152.67 ± 63.17	171.43 ± 60.51	194.36 ± 47.50
P06	184.71 ± 46.24	150.91 ± 35.63	166.45 ± 47.22	231.68 ± 72.13
P07	161.31 ± 41.08	213.88 ± 72.95	219.44 ± 50.37	256.59 ± 96.18
P08	137.38 ± 25.55	126.53 ± 21.29	165.45 ± 32.09	164.97 ± 30.31
P09	230.88 ± 31.15	243.88 ± 106.96	250.42 ± 40.36	254.76 ± 43.44
P10	182.30 ± 46.90	293.21 ± 62.44	204.48 ± 50.39	244.16 ± 68.07
P11	284.10 ± 54.54	267.96 ± 54.29	436.41 ± 81.61	411.35 ± 79.42
P12	304.70 ± 53.23	294.60 ± 60.72	340.10 ± 47.46	341.41 ± 50.35
P13	312.36 ± 59.01	310.25 ± 62.05	337.44 ± 45.57	336.99 ± 54.16
P14	228.42 ± 102.32	261.10 ± 78.63	272.45 ± 71.13	244.62 ± 77.65
P15	308.40 ± 43.48	320.46 ± 52.46	385.39 ± 54.75	319.07 ± 53.92
P16	123.19 ± 18.73	119.13 ± 18.36	125.52 ± 19.98	152.52 ± 32.03
Mean	241.18 ± 87.10	252.72 ± 85.96	288.60 ± 119.34	294.50 ± 104.94

Supplementary Table 3. Post-hoc pairwise comparisons between each pair of task movement durations using Wilcoxon signed-rank tests.

	Z	*p*
Thumb vs Index	-1.0859	0.3330
Thumb vs Middle	-3.4128	0.0019
Thumb vs Little	-3.5162	0.0019
Index vs Middle	-2.7923	0.0105
Index vs Little	-2.6371	0.0125
Middle vs Little	-0.6722	0.5015

Supplementary Table 4. Classification accuracy using EMG signals.

Participant	Accuracy
P01	76.50%
P02	90.25%
P03	78.50%
P04	83.50%
P05	79.25%
P06	77.50%
P07	90.50%
P08	68.75%
P09	89.75%
P10	84.00%
P11	95.50%
P12	68.75%
P13	90.00%
P14	78.50%
P15	93.75%
P16	83.25%
Mean	83.02%
SD	8.20%

Supplementary Table 5. Classification accuracy using PCA-based method across all frequency bands.

Participant	*δ*	*θ*	*α*	*β*	*γ*	high-*γ*
P01	37.75%	33.75%	25.50%	28.25%	22.25%	25.00%
P02	54.75%	36.75%	28.75%	27.25%	22.75%	25.25%
P03	45.00%	32.75%	26.75%	19.25%	21.50%	24.00%
P04	37.00%	39.00%	20.25%	25.00%	26.50%	24.25%
P05	49.00%	40.50%	32.25%	22.50%	27.00%	25.50%
P06	44.00%	31.00%	23.50%	25.50%	25.25%	23.25%
P07	51.50%	34.25%	25.75%	25.50%	24.75%	26.00%
P08	47.50%	33.00%	29.75%	21.00%	24.50%	27.25%
P09	40.75%	32.50%	26.75%	23.50%	21.75%	28.75%
P10	41.00%	28.50%	25.25%	25.25%	25.00%	27.25%
P11	50.50%	35.50%	23.25%	24.25%	26.00%	24.25%
P12	44.00%	31.75%	25.00%	27.00%	28.25%	26.25%
P13	52.00%	30.50%	29.00%	25.25%	27.25%	24.00%
P14	59.00%	43.00%	30.25%	22.75%	29.50%	25.25%
P15	48.00%	40.75%	31.00%	27.00%	26.75%	26.00%
P16	37.75%	27.75%	31.00%	26.00%	19.50%	24.75%
Mean	46.22%	34.45%	27.13%	24.70%	24.91%	25.44%
SD	6.46%	4.48%	3.34%	2.41%	2.73%	1.44%

Supplementary Table 6. Classification accuracy using spatial locations derived from full-epoch activation index maps across all frequency bands.

Participant	*δ*	*θ*	*α*	*β*	*γ*	high-*γ*
P01	52.25%	46.00%	31.75%	23.50%	24.25%	24.50%
P02	62.75%	40.50%	32.25%	30.25%	26.00%	22.25%
P03	45.75%	33.50%	26.25%	23.75%	27.50%	24.00%
P04	52.50%	36.00%	26.00%	24.25%	24.75%	25.75%
P05	46.75%	42.00%	34.00%	25.75%	26.50%	24.50%
P06	48.50%	35.25%	27.00%	24.50%	23.50%	24.00%
P07	54.00%	36.50%	26.50%	25.75%	31.00%	27.25%
P08	52.75%	42.50%	31.00%	25.75%	23.25%	24.50%
P09	50.50%	34.25%	23.50%	21.25%	25.75%	28.75%
P10	48.50%	29.25%	31.25%	25.00%	29.00%	24.50%
P11	63.25%	35.00%	30.25%	32.00%	27.25%	25.00%
P12	48.50%	35.25%	24.00%	23.75%	29.50%	28.25%
P13	64.00%	38.00%	27.75%	25.00%	28.25%	22.00%
P14	73.75%	40.00%	33.75%	25.50%	26.25%	25.75%
P15	68.00%	52.50%	34.00%	21.25%	23.75%	29.00%
P16	50.00%	38.00%	21.00%	23.25%	24.00%	27.00%
Mean	55.11%	38.41%	28.77%	25.03%	26.28%	25.44%
SD	8.47%	5.53%	4.07%	2.78%	2.33%	2.12%

Supplementary Table 7. Classification accuracy using spatial locations derived from short-window activation index maps across all frequency bands.

Participant	*δ*	*θ*	*α*	*β*	*γ*	high-*γ*
P01	58.00%	47.75%	30.50%	26.50%	29.25%	26.00%
P02	70.50%	36.75%	31.50%	26.50%	21.25%	24.00%
P03	54.00%	37.00%	30.00%	22.75%	28.25%	23.25%
P04	57.25%	34.75%	23.50%	26.75%	24.25%	26.00%
P05	59.25%	45.25%	35.50%	28.25%	21.75%	25.00%
P06	49.75%	38.00%	29.50%	26.25%	23.25%	23.00%
P07	59.25%	41.00%	28.50%	26.50%	33.75%	27.25%
P08	57.00%	39.25%	36.00%	23.50%	24.50%	29.00%
P09	61.75%	34.00%	20.75%	25.75%	23.50%	26.75%
P10	53.25%	30.25%	26.25%	23.50%	25.75%	21.75%
P11	62.00%	40.25%	28.25%	25.25%	25.50%	23.50%
P12	55.50%	42.00%	21.00%	22.50%	28.00%	28.50%
P13	63.00%	42.75%	35.00%	25.50%	32.00%	22.50%
P14	71.50%	51.00%	32.50%	27.00%	31.00%	25.75%
P15	72.50%	51.25%	37.75%	22.75%	24.00%	25.00%
P16	52.50%	38.00%	26.75%	25.75%	25.00%	27.00%
Mean	59.81%	40.58%	29.58%	25.31%	26.31%	25.27%
SD	6.83%	5.94%	5.14%	1.76%	3.69%	2.14%

Supplementary Table 8. Post-hoc pairwise comparisons between classification accuracies across frequency bands and chance-level using Wilcoxon signed-rank tests.

	**Z**	***p***
δ - PCA	3.5174	0.0013
δ - *A*-epoch	3.5185	0.0013
δ - *A*-window	3.5168	0.0013
θ - PCA	3.5162	0.0013
θ - *A*-epoch	3.5174	0.0013
θ - *A*-window	3.5168	0.0013
α - PCA	/	0.0444
α - *A*-epoch	2.7937	0.0134
α - *A*-window	2.7406	0.0138
β - PCA	/	0.9008
β - *A*-epoch	/	0.6777
β - *A*-window	0.5190	0.7245
γ - PCA	/	0.9008
γ - *A*-epoch	1.8110	0.1263
γ - *A*-window	/	0.5483
high-*γ* - PCA	/	0.5274
high-*γ* - *A*-epoch	/	0.6777
high-*γ* - *A*-window	/	0.7929

Supplementary Table 9. Post-hoc pairwise comparisons of classification accuracies across different frequency bands.

Contrast	estimate	SE	df	t.ratio	*p*
δ vs θ	49.22	12.1	75	4.080	0.0015
δ vs α	128.92	12.1	75	10.688	<0.0001
δ vs β	184.78	12.1	75	15.319	<0.0001
δ vs γ	170.58	12.1	75	14.142	<0.0001
δ vs high-γ	178.12	12.1	75	14.767	<0.0001
θ vs α	79.7	12.1	75	6.607	<0.0001
θ vs β	135.56	12.1	75	11.239	<0.0001
θ vs γ	121.36	12.1	75	10.061	<0.0001
θ vs high-γ	128.91	12.1	75	10.687	<0.0001
α vs β	55.86	12.1	75	4.631	0.0002
α vs γ	41.67	12.1	75	3.454	0.0113
α vs high-γ	49.21	12.1	75	4.080	0.0015
β vs γ	-14.2	12.1	75	-1.177	0.8463
β vs high-γ	-6.66	12.1	75	-0.552	0.9937
γ vs high-γ	7.54	12.1	75	0.625	0.9888

Supplementary Table 10. Post-hoc pairwise comparisons of classification accuracies with different methods.

Contrast	estimate	SE	df	t.ratio	*p*
PCA vs *A*-epoch	-49.6	6.88	30	-7.213	<0.0001
PCA vs *A*-window	-78.7	6.88	30	-11.445	<0.0001
A-epoch vs *A*-window	-29.1	6.88	30	-4.232	0.0006

Supplementary Table 11. Post-hoc pairwise comparisons of classification accuracies using different methods within each frequency bands.

Contrast	estimate	SE	df	t.ratio	*p*
δ, PCA vs *A*-epoch	-52.81	19.2	180	-2.749	0.018
δ, PCA vs *A*-window	-124.5	19.2	180	-6.481	<0.0001
δ, *A*-epoch vs *A*-window	-71.69	19.2	180	-3.732	0.0007
θ, PCA vs *A*-epoch	-17.06	19.2	180	-0.888	0.6485
θ, PCA vs *A*-window	-33.75	19.2	180	-1.757	0.1872
θ, *A*-epoch vs *A*-window	-16.69	19.2	180	-0.869	0.6607
α, PCA vs *A*-epoch	23.31	19.2	180	1.214	0.4467
α, PCA vs *A*-window	30.75	19.2	180	1.601	0.248
α, *A*-epoch vs *A*-window	7.44	19.2	180	0.387	0.9207
β, PCA vs *A*-epoch	59.88	19.2	180	3.117	0.006
β, PCA vs *A*-window	83.12	19.2	180	4.327	0.0001
β, *A*-epoch vs *A*-window	23.25	19.2	180	1.210	0.4486
γ, PCA vs *A*-epoch	32.06	19.2	180	1.669	0.22
γ, PCA vs *A*-window	64.25	19.2	180	3.345	0.0029
γ, *A*-epoch vs *A*-window	32.19	19.2	180	1.676	0.2174
high-*γ*, PCA vs *A*-epoch	67.06	19.2	180	3.491	0.0017
high-*γ*, PCA vs *A*-window	104.5	19.2	180	5.440	<0.0001
high-*γ*, *A*-epoch vs *A*-window	37.44	19.2	180	1.949	0.1282

Supplementary Table 12. Classification accuracy using features from different windows for the δ-band.

Participant	Win-comb	Win -200~-150 ms	Win -150~-100 ms	Win -100~-50 ms	Win -50~0 ms	Win 0~50 ms	Win 50~100 ms	Win 100~150 ms	Win 150~200 ms	Win 200~250 ms	Win 250~300 ms	Win 300~350 ms	Win 350~400 ms	Win 400~450 ms	Win 450~500 ms	Win 500~550 ms	Win 550~600 ms	Win 600~650 ms	Win 650~700 ms	Win 700~750 ms
P01	65.25%	55.00%	52.00%	54.50%	56.75%	56.50%	48.25%	56.25%	58.00%	54.50%	58.00%	59.75%	57.00%	47.25%	51.75%	53.25%	50.25%	44.75%	38.00%	57.75%
P02	80.75%	71.75%	65.75%	69.00%	66.50%	66.25%	66.25%	63.75%	58.50%	58.25%	58.50%	66.50%	60.00%	61.25%	65.75%	68.50%	66.75%	58.00%	70.50%	68.75%
P03	67.00%	53.25%	58.50%	53.50%	53.75%	52.00%	55.50%	61.25%	49.00%	57.00%	53.75%	51.25%	50.25%	51.00%	56.75%	50.75%	56.25%	54.75%	59.50%	56.50%
P04	63.50%	51.50%	51.50%	53.50%	53.00%	53.75%	55.75%	54.25%	48.50%	48.50%	41.50%	44.75%	43.00%	44.75%	43.25%	47.75%	47.50%	53.00%	47.00%	51.25%
P05	64.25%	58.75%	56.25%	55.25%	59.00%	54.50%	57.75%	54.00%	55.75%	57.50%	60.00%	56.50%	54.75%	52.00%	57.75%	58.50%	59.25%	58.00%	58.75%	57.25%
P06	57.75%	45.00%	54.25%	45.50%	45.75%	44.50%	49.50%	45.00%	42.50%	40.75%	43.75%	39.25%	46.00%	44.00%	38.50%	48.50%	48.25%	44.75%	46.25%	47.50%
P07	72.00%	60.50%	58.00%	55.75%	60.00%	59.50%	57.25%	54.25%	61.00%	59.00%	58.00%	55.50%	56.25%	58.00%	54.00%	57.00%	54.75%	56.50%	54.50%	52.75%
P08	64.00%	53.25%	56.75%	54.75%	55.50%	54.00%	50.75%	53.25%	52.50%	52.00%	50.75%	49.50%	45.50%	51.75%	47.00%	49.25%	56.00%	53.00%	41.50%	50.00%
P09	72.25%	52.75%	56.50%	51.75%	44.25%	52.50%	55.00%	43.50%	48.25%	53.50%	47.25%	44.50%	42.75%	51.75%	53.50%	49.25%	43.50%	45.25%	51.75%	54.00%
P10	61.75%	51.50%	48.25%	40.00%	53.25%	51.50%	52.50%	47.50%	48.00%	47.25%	51.75%	42.75%	39.75%	53.25%	54.25%	48.25%	49.25%	48.50%	51.00%	53.50%
P11	75.00%	60.00%	58.75%	55.00%	55.25%	62.50%	65.75%	57.75%	61.00%	52.75%	54.75%	56.50%	49.25%	52.25%	52.00%	58.75%	56.50%	55.75%	48.75%	54.50%
P12	68.00%	58.50%	60.75%	58.00%	49.75%	59.75%	57.00%	55.75%	55.75%	56.25%	57.00%	57.50%	54.75%	54.75%	56.50%	54.25%	53.75%	55.50%	50.75%	59.75%
P13	67.75%	57.75%	58.50%	64.75%	66.25%	65.00%	63.25%	59.00%	57.25%	62.00%	54.00%	44.50%	48.25%	50.50%	56.75%	56.00%	54.75%	47.25%	62.25%	60.25%
P14	85.75%	67.75%	67.75%	69.00%	65.00%	68.75%	70.75%	66.75%	60.25%	65.75%	57.75%	59.25%	62.25%	63.25%	57.75%	68.50%	62.50%	63.00%	61.00%	64.75%
P15	82.00%	72.75%	72.00%	78.00%	71.00%	74.75%	70.75%	72.25%	69.25%	65.00%	58.50%	70.75%	67.25%	67.75%	70.50%	63.00%	65.75%	73.50%	70.25%	75.75%
P16	58.75%	47.00%	46.75%	43.00%	49.75%	48.50%	50.75%	48.75%	53.25%	48.00%	45.50%	43.75%	47.50%	49.25%	52.00%	45.75%	52.75%	48.50%	50.00%	52.00%
Mean	69.11%	57.31%	57.64%	56.33%	56.55%	57.77%	57.92%	55.83%	54.92%	54.88%	53.17%	52.66%	51.53%	53.30%	54.25%	54.83%	54.86%	53.75%	53.86%	57.27%
SD	8.27%	8.01%	6.73%	9.91%	7.68%	8.03%	7.33%	7.74%	6.68%	6.70%	5.86%	9.09%	7.73%	6.47%	7.65%	7.18%	6.47%	7.57%	9.29%	7.35%

Supplementary Table 13. Classification accuracy using features from different windows for the *θ*-band.

Participant	Win-comb	Win -100~-50 ms	Win -50~0 ms	Win 0~50 ms	Win 50~100 ms	Win 100~150 ms	Win 150~200 ms	Win 200~250 ms	Win 300~350 ms	Win 350~400 ms	Win 400~450 ms	Win 450~500 ms	Win 500~550 ms	Win 550~600 ms
P01	57.00%	34.75%	51.00%	48.75%	55.50%	49.75%	56.75%	54.00%	59.00%	58.75%	49.50%	51.25%	49.75%	50.25%
P02	49.00%	40.00%	42.25%	41.00%	39.50%	41.50%	39.00%	37.25%	40.75%	38.75%	40.75%	41.75%	35.50%	41.50%
P03	39.00%	40.00%	36.00%	40.00%	41.00%	42.75%	42.00%	41.25%	33.50%	38.00%	42.75%	38.00%	40.00%	39.75%
P04	37.00%	39.25%	38.25%	38.75%	38.00%	39.25%	37.25%	37.75%	37.25%	34.75%	30.50%	35.00%	38.75%	39.25%
P05	44.00%	50.00%	52.00%	49.50%	50.50%	54.50%	54.00%	48.25%	50.00%	47.75%	49.25%	49.50%	51.75%	50.25%
P06	49.50%	40.00%	40.25%	44.00%	45.50%	41.00%	45.50%	42.75%	41.75%	44.00%	41.75%	42.50%	40.75%	32.75%
P07	48.00%	37.50%	43.25%	31.75%	38.00%	43.75%	38.25%	37.50%	37.50%	37.00%	37.75%	38.75%	43.50%	41.00%
P08	47.00%	39.00%	51.25%	51.00%	50.50%	53.25%	53.75%	54.75%	53.75%	48.25%	53.50%	54.25%	51.50%	52.25%
P09	44.50%	40.50%	45.75%	47.75%	47.00%	47.00%	37.75%	44.50%	40.50%	39.75%	37.00%	38.50%	30.25%	32.75%
P10	39.00%	31.50%	38.75%	33.50%	37.50%	34.25%	30.25%	31.00%	25.75%	27.25%	23.00%	31.75%	24.75%	27.00%
P11	43.50%	32.50%	45.25%	41.25%	46.50%	47.00%	45.00%	45.50%	43.50%	43.50%	37.25%	38.50%	44.50%	42.50%
P12	46.75%	40.50%	43.50%	45.50%	45.75%	47.50%	45.75%	45.75%	47.00%	47.75%	46.50%	45.75%	46.50%	48.25%
P13	46.75%	40.75%	43.75%	49.50%	46.00%	44.75%	47.25%	46.50%	46.75%	49.00%	47.50%	45.25%	48.25%	44.00%
P14	56.75%	39.50%	44.75%	49.75%	55.50%	57.75%	50.00%	43.50%	51.25%	56.00%	51.25%	44.75%	53.25%	41.25%
P15	61.25%	63.00%	65.50%	66.50%	67.00%	67.75%	66.75%	68.00%	68.75%	67.75%	67.25%	69.00%	66.75%	70.00%
P16	44.25%	39.50%	44.50%	43.75%	44.25%	40.25%	40.25%	42.00%	41.75%	45.25%	43.50%	42.50%	38.50%	38.50%
Mean	47.08%	40.52%	45.38%	45.14%	46.75%	47.00%	45.59%	45.02%	44.92%	45.22%	43.69%	44.19%	44.02%	43.20%
SD	6.70%	7.26%	7.05%	8.11%	7.85%	8.24%	9.08%	8.62%	10.29%	9.90%	10.11%	8.88%	9.98%	9.89%

Supplementary Table 14. Post-hoc pairwise comparisons of *δ*-band classification accuracies the window-combined approach and all single-window conditions using Wilcoxon signed-rank tests.

	**Z**	***p***
Window-combined vs Window -200~-150 ms	3.5168	0.0044
Window-combined vs Window -150~-100 ms	3.5174	0.0044
Window-combined vs Window -100~-50 ms	3.5168	0.0044
Window-combined vs Window -50~0 ms	3.5191	0.0044
Window-combined vs Window 0~50 ms	3.5180	0.0044
Window-combined vs Window 50~100 ms	3.5168	0.0044
Window-combined vs Window 100~150 ms	3.5162	0.0044
Window-combined vs Window 150~200 ms	3.5162	0.0044
Window-combined vs Window 200~250 ms	3.5174	0.0044
Window-combined vs Window 250~300 ms	3.5191	0.0044
Window-combined vs Window 300~350 ms	3.5168	0.0044
Window-combined vs Window 350~400 ms	3.5162	0.0044
Window-combined vs Window 400~450 ms	3.5168	0.0044
Window-combined vs Window 450~500 ms	3.5168	0.0044
Window-combined vs Window 500~550 ms	3.5168	0.0044
Window-combined vs Window 550~600 ms	3.5162	0.0044
Window-combined vs Window 600~650 ms	3.5174	0.0044
Window-combined vs Window 650~700 ms	3.5168	0.0044
Window-combined vs Window 700~750 ms	3.5174	0.0044

Supplementary Table 15. Post-hoc pairwise comparisons of *θ*-band classification accuracies the window-combined condition and all single-window conditions using Wilcoxon signed-rank tests.

	**Z**	***p***
Window-combined vs Window -100~-50 ms	2.8703	0.0555
Window-combined vs Window -50~0 ms	1.1125	0.5239
Window-combined vs Window 0~50 ms	1.0866	0.5255
Window-combined vs Window 50~100 ms	/	0.9663
Window-combined vs Window 100~150 ms	0.2068	0.9285
Window-combined vs Window 150~200 ms	0.8801	0.6276
Window-combined vs Window 200~250 ms	/	0.5239
Window-combined vs Window 300~350 ms	/	0.4749
Window-combined vs Window 350~400 ms	/	0.6616
Window-combined vs Window 400~450 ms	2.0694	0.2503
Window-combined vs Window 450~500 ms	1.8113	0.3179
Window-combined vs Window 500~550 ms	1.3192	0.4152
Window-combined vs Window 550~600 ms	1.7581	0.3179
